# Developing a Health App Quality Report for CEN-ISO/TS 82304-2 to Satisfy Health Care Professionals’ Information Needs on the Quality of Health Apps: Participatory Design Study

**DOI:** 10.2196/86473

**Published:** 2026-10-23

**Authors:** Petra Hoogendoorn, Antanas Montvila, Kristina Ziuteliene, Paulius Algirdas Povilonis, María Villalobos-Quesada, Niels H Chavannes, Maaike Kleinsmann, Jochen Klucken

**Affiliations:** 1 National eHealth Living Lab, Department of Public Health and Primary Care, Leiden University Medical Center, Albinusdreef 2, 2333 ZA Leiden, The Netherlands, 31 654341785; 2 Radiology Department, The Hospital of Lithuanian University of Health Sciences, Kaunas Clinics, Kaunas, Lithuania; 3 Department of Family Medicine, Lithuanian University of Health Sciences, Kaunas, Lithuania; 4 Department of Design, Organisation and Strategy, Faculty of Industrial Design Engineering, Delft University of Technology, Delft, The Netherlands; 5 Digital Medicine, Luxemburg Centre for Systems Biomedicine, University of Luxembourg, Belvaux, Luxembourg; 6 Digital Medicine Group, Centre Hospitalier de Luxembourg, Strassen, Luxembourg

**Keywords:** health app, digital health, health care providers, medical societies, information needs, participatory design

## Abstract

**Background:**

Health apps, which comprise medical and wellness apps, hold potential to improve prevention, diagnosis, treatment, and management of disease. Yet, recommendation and prescription rates by health care professionals (HCPs) are low. Trusted standardized health app quality reports providing the information HCPs need to recommend or prescribe a specific app for an individual patient and guidelines from medical societies are considered crucial to enable this digital transformation of medicine. The CEN-ISO 82304‐2 (hereinafter “82304‐2”) Technical Specification, an initiative of the European Commission, includes a research-based health app quality assessment framework comprising 81 quality requirements. Results of 82304‐2 app assessments are summarized in an intuitive “health app quality label.” The 82304‐2 quality label’s potential to increase HCP willingness to recommend health apps was recently confirmed. To adequately enable decision-making and thus app recommendations, 82304‐2 proposes a more detailed “health app quality report” to complement the 82304‐2 quality label.

**Objective:**

This study aimed to design the 82304‐2 health app quality report that satisfies the information needs of individual HCPs in decision-making on an individual health app for an individual patient.

**Methods:**

A participatory design approach was applied. In an 18-month process of participatory prototyping with 9 HCPs with digital health expertise, the 82304‐2 quality report was iteratively designed. A convenience sample of 31 HCPs indicated the priority 82304‐2 quality requirements they considered useful in decision-making on recommending an app, and thus needed detailed quality information. Final feedback meetings with the 9 HCPs and 8 medical societies were used to finalize the 82304‐2 quality report design. Web-based questions in these meetings, feedback from the Dutch Patient Federation, and a comparative content analysis and the Health Education Materials Assessment Tool were used to evolve to and evaluate the final 82304‐2 quality report design.

**Results:**

In total, 37% (30/81) of the 82304‐2 quality requirements were prioritized by >50% of the HCPs. The final 82304‐2 quality report design provides detailed information for 90% (27/30) of the prioritized quality requirements. The reporting detail for 2 quality requirements requires more research. The final feedback meetings, the Patient Federation feedback, comparative content analysis, and Health Education Materials Assessment Tool provide indicative proof of the usefulness and usability of the quality report design.

**Conclusions:**

We succeeded in our aim to design the 82304‐2 health app quality report and found promising potential for its distinctive usefulness for HCPs and patients in recommending and considering a health app, respectively, and for medical societies in creating guidance. Further efforts are needed to test its effectiveness, fine-tune multistakeholder and intercontinental usefulness and usability, support medical societies in providing guidance and potentially training on recommending health apps, and advance from the current design to a scalable fully functional version of the quality report and associated open access database.

## Introduction

Within the broader field of digital health technologies, health apps are defined as software applications that can be executed on a computing platform, intended specifically for managing, maintaining, or improving the health of individual persons or the delivery of health care [1]. Health apps comprise both apps that can be used for medical purposes and wellness apps [2]. Patients’ willingness to use health apps is high [3]. Recommendations provided by their health care professionals (HCPs) positively affect patients’ trust in and willingness to use, pay for, and download a health app, and matter especially for those with lower digital literacy [4-8]. Only some countries have already implemented reimbursement mechanisms for health apps that require app prescriptions by HCPs [9,10]. Germany, France, and Belgium are recognized front-runners [9]. Germany now reimburses 57 apps via its “DiGA” fast-track and France 4 apps via its “PECAN” initiative [11]. Belgium has exchanged its individual app (“validation pyramid”) approach for a care pathway approach where participating hospitals individually identify, evaluate, select, and, if applicable, procure the type of apps described in their contract with the funding agency. The first care pathway (telemonitoring after hospitalization for heart failure) is now approved [11]. Of these 3 countries, Germany is the only one to provide HCPs with a directory with detailed information on the prescribable reimbursed health apps [11], all of which are evidence-validated digital medical devices, referred to in German as “Digitale Gesundheitsanwendungen” or “DiGA” [12].

German prescription behavior studies indicate that HCPs increasingly observe benefits of these apps, such as health promotion, mobility, prevention, empowerment, health literacy, improved adherence, disease self-management, health benefits, and better access to care. Yet, intent to prescribe or recommend them and the number of actual prescriptions are low and differ per medical specialty [13-18]. Over the course of 5 years on a population of 73 million in statutory health insurance, in total 1.6 million app prescriptions have been issued by German HCPs, predominantly (51%) by general practitioners (GPs) [19,20]. Yet, as a recent study with German GPs (n=5868) shows, even within this group, confidence in ability to give patients capable advice on DiGA is low. Especially, GPs from rural areas report a lack of this confidence (urban 42% and rural 9%). They also report less often a favorable opinion of apps (urban 92% and rural 29%) and confidence in the DiGA directory (92% urban and 30% rural). Overall, 14% of GPs had prescribed DiGA (21% urban and 5% rural), and 13% planned to prescribe them in the near future [18]. For Belgium and France neither prescription numbers nor prescription behavior studies were found [11].

A recent systematic review demonstrated the potential of interventions to encourage app prescriptions by HCPs, with 91% of studies (10/11) reporting positive results, although sample sizes and follow-up were generally limited [21]. The range of interventions studied did not include structured reports on the quality of a given health app, nor guidelines from medical professional societies (hereinafter “medical societies”), despite a distinct need for and benefit of this type of information and guidance [3,7,13,14,16-18,22]. For example, Canadian HCPs (n=117) prioritized a health app quality report (“unbiased standardized review by experts”) (80/117, 68%) and guidelines (72/117, 62%) as sources of information for recommending or prescribing apps [5]. German diabetes HCPs (n=538) expressed a willingness to include apps in their type 2 diabetes prevention, monitoring, and management far more (26%) or rather more (54%) if the national guideline would address the use of apps in these areas [16]. The European Society of Cardiology recently confirmed a need for accepted criteria for uptake of apps in guidelines and for standardized assessment, referencing CEN-ISO/TS 82304‐2 for its potential to become a widely used and efficient tool to help drive health app recommendations internationally [23].

CEN-ISO/TS 82304-2:2021 Health software Part 2: Health and wellness apps—Quality and reliability [1] (hereinafter “82304‐2”) was an assignment from the European Commission to the European Committee for Standardization (CEN). The initiative became a global standardization effort due to its collaboration with the International Organization for Standardization (ISO) and the International Electrotechnical Commission (IEC). 82304‐2 provides a health app quality assessment framework and a health app quality label. The quality assessment framework was built on a Delphi study with 83 experts from 8 stakeholder groups, including HCPs and medical organizations, and consists of 81 health app quality requirements [24]. Sixty-seven of these impact 4 overarching scores, which were named “Healthy and safe,” “Easy to use,” “Secure data,” and “Robust build” after testing with persons with low health literacy. The 82304‐2 quality label summarizes the results of an 82304‐2 quality assessment for an individual app in these 4 scores in a simple and intuitive way, with comparative discrete A-E scales, traffic light color coding, and score bar length.

82304‐2 also introduces a health app quality report. While the label displays the results of the 4 overarching scores, the report is described as the set of answers to all 81 quality requirements and by its aims to enable multistakeholder informed decision-making on the quality-assessed app. Whereas the label is specified in detail (Figure 1), 82304-2 does not provide a design of the quality report, nor a specification of the granularity of information within it [1].

**Figure 1.**
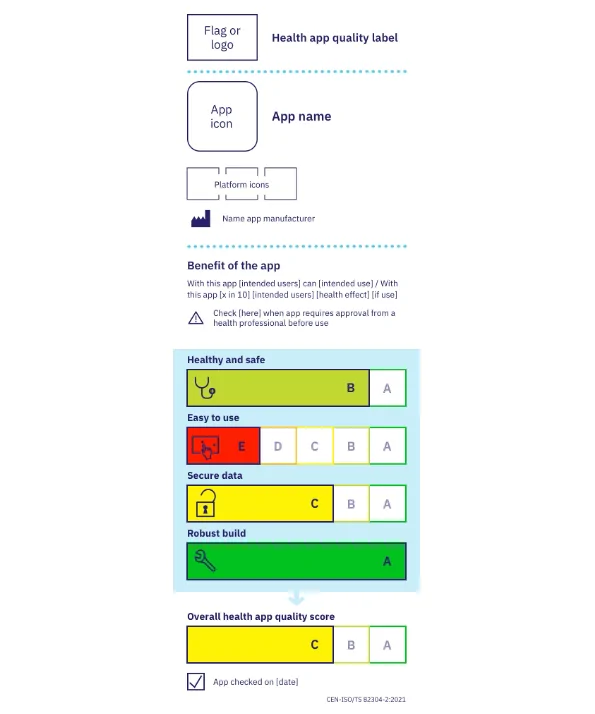
CEN-ISO/TS 82304‐2 health app quality label design.

An experimental vignette study (n=116) confirmed the potential of the 82304‐2 quality label to increase HCP willingness to recommend a range of health apps (prevention, self-management, and health care related) to a range of patients (low and high socioeconomic status). Moreover, the label positively affected “Acceptability,” the criterion most strongly correlated to willingness to recommend health apps. The study underlined that once developed, the more detailed 82304‐2 quality report may further increase HCP willingness to recommend high-quality apps [25]. A follow-up study (n=290) explored what HCPs would need beyond the 82304‐2 quality label and found that increasing knowledge, boosting confidence, and forming habits had the highest potential for change in HCP health app recommendation behavior [26]. Unfortunately, the research method did not provide a specification of “knowledge” and as such direction for the type of information and granularity HCPs would need in the 82304‐2 health app quality report. With regard to citizens (patients), separate yet to be published studies found a positive effect of the 82304‐2 quality label on their intent to download a health app (n=1105) and their ability to choose “the right app” with the label and a short educational video to explain the label (n=789). Both studies took measures to ensure inclusion of persons with low health literacy.

To our knowledge, literature on the information detail that individual HCPs would need to make informed decisions about recommending the right health app to the right patient and report designs that support such decision-making is scarce. The only sources we found indicated that even the detailed information provided by Germany’s national directory of certified DiGA [12] was perceived as not detailed enough and lacking well-founded reliable information [17,18]. Especially information on functionalities and (research about) benefits and risks was considered not sufficient [22]. A user survey and routine data analysis by a German insurer proposed to improve the DiGA directory by adding sufficiently detailed information about the range of indications and contraindications, including age and gender, and a brief uniform understandable explanation of the app content and key functionalities within a few clicks [27].

Given the importance of HCPs for decision-making by their patients, the objective of this study is to design the 82304‐2 quality report that satisfies the information needs of individual HCPs in decision-making on recommending the app covered by the quality report to an individual patient. We aim to uncover which 82304‐2 quality requirements need which specific information in which granularity, and which graphical design is expected to support HCP decision-making best, effectively pursuing usefulness and usability and, as such, use of the 82304‐2 quality report by HCPs. Although our aim is to expand the usefulness and usability of the quality report to more stakeholders over time, our focus in this study is limited to HCPs and the potential compatibility of the resulting quality report with the information needs of medical societies and patients.

## Methods

### Participatory Design

Participatory design (PD) is a user-centered design method that actively involves future users as co-designers in generally an iterative design process in which changes to the design based on feedback by users are applied between design sessions [28-30]. This user involvement increases the likelihood that the design fits user needs and will be adopted and used as intended [30]. PD is particularly helpful for developing health information visualizations, that is, text and images with a communicative intent that aim to improve health outcomes by means of improved comprehension, communication, or engagement, and subsequent behavior changes [28]. PD is frequently used in health informatics and has been proven capable of changing clinical practice [28,31]. Since its inception in the late 1970s in Scandinavia in the computer science field, PD has evolved significantly. A recent systematic review showed that users are generally involved throughout a design process in which multiple techniques are used. The techniques can either be predetermined or emergent [29]. Our approach was emergent.

### Methodological Elements and Principles

#### Overview

PD methods are typically tailored to the project-specific context [32]. Methodological elements that play a key role in PD research are (1) recruitment and management of stakeholders, (2) the use of tools and techniques, and (3) outcome measures [29,33]. PD principles that can aid in methodological decision-making include (1) democracy, the aim to involve all stakeholders including nondesigners and future users to achieve diversity of experience, values, and knowledge; (2) mutual learning; (3) tacit or latent needs assessment, transcending the easily detectable needs; and (4) collective creativity, in which values emerge and become inscribed in the product [33]. To achieve a comprehensive, useful, and usable 82304‐2 quality report, these methodological elements were tailored—aided by the PD principles—as follows.

#### Recruitment and Management of Stakeholders

For the project as a whole, the Label2Enable HCP Advisory Board (in short, HCP Advisory Board) was set up to ensure a comprehensive representation of HCPs, contributing to the effectiveness and impact of several project tasks, yet with a focus on being co-designers in this design study. A committee of user representatives is one of the PD techniques identified by Wacnik et al [29]. According to the grant agreement, the HCP Advisory Board was to consist of individuals representing different specialties, medical organizations, clinical settings, age groups, and nationalities to ensure sufficient diversity. To ultimately achieve the PD principle “collective creativity,” AM and KZ added as a requirement digital health expertise. Expertise and diversity are recognized to positively affect PD [33]. Recruitment was achieved by purposive sampling, using existing networks, and identifying and contacting European medical societies via email. Eleven HCPs with expertise were shortlisted, and 9 agreed to join the HCP Advisory Board. See Multimedia Appendix 1 for the HCP Advisory Board. The size of the HCP Advisory Board resulted from our aims to achieve sufficient expertise and diversity while fostering engagement [28]. All the HCP Advisory Board meetings were in English and online, given the various native languages and geographic locations. Members of the HCP Advisory Board received symbolic remuneration for their efforts. A diverse representation of medical societies was invited to assist in connecting to the medical community at large and ultimately to participate in final feedback meetings. The Label2Enable Patient Advisory Board was consulted once during the PD process and 2 employees of the Dutch Patient Federation provided feedback from a patient perspective on the final design of the health app quality report.

#### Tools and Techniques

##### Overview

According to Vandekerckhove et al [33], depending on the aim of PD, tools can be used to (1) inspire ideas, (2) immerse participants in a domain, (3) gain an understanding, and (4) generate new ideas. The tools used in this study were participatory prototyping, final feedback meetings, a comparative content analysis, and the Health Education Materials Assessment Tool (HEMAT) (Figure 2). Their purpose, as distinguished by Vandekerckhove et al [33], and the PD techniques used, as classified by Wacnik et al [29], are further detailed in the following paragraphs . Although Wacnik et al [29] provided high-level definitions for the PD techniques distinguished, the authors stressed that techniques can, and in the spirit of adaptability should, stray from these definitions.

**Figure 2.**
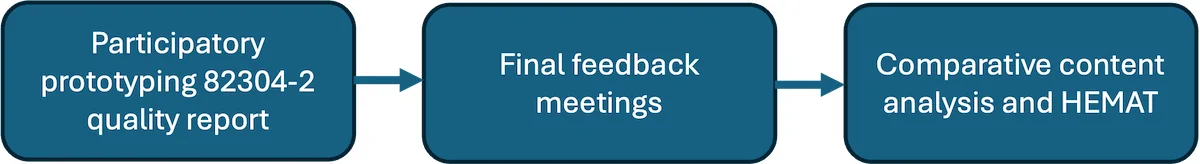
Graphic overview of the iterative stages of this participatory design study. HEMAT: Health Education Materials Assessment Tool.

##### Participatory Prototyping 82304‐2 Quality Report

First, to inspire ideas, PH searched online for designs of what could be described as quality reports for health apps and other products that HCPs and other stakeholder groups may be familiar with in the European context (PD technique: competitive benchmarking). This aligns with the Data-Frame Theory of Sensemaking, which suggests that people make sense of data by comparing them with frames from prior lived experiences, easing sensemaking. Stoplight colors to convey value judgments are a well-known example [28]. The quality reports analyzed, with in-between brackets the type of products they report on, included the European Product Registry for Energy Labelling database (European Union [EU] energy-labeled products) [34], the German DiGA directory (digital health applications) [12], the Finnish Digi-HTA directory (digital products and services for social and health care and well-being) [35], and the Summary of Product Characteristics, commonly known as SmPC (medicinal products) [36]. Later, additionally, the AppGuide (all health and wellness apps in the App Store and Google Play Store) [37], the European Database on Medical Devices [38], and the International HTA database (Health Technology Assessments from 53 HTA agencies from 33 countries around the globe) [39] were examined. These quality reports were where considered relevant evaluated with AM and graphic designer Oscar Smeulders and inspired the 82304‐2 quality report prototyping, similar to how the designs of the EU energy label [40] and Nutri-Score front-of-pack nutrition label [41], and the content of the United States Food and Drug Administration over-the-counter medicine label [42] inspired the 82304‐2 quality label design and content.

Second, to immerse participants, PH used a PowerPoint presentation to make the HCP Advisory Board acquainted with the 82304‐2 quality assessment framework and quality label and exemplary quality report designs found in the online search to provide a sense of direction for this study (PD technique: participatory workshop). Initial stimuli to respond to are found to be more effective in PD than presenting a blank slate [28]. While the exemplary quality reports provided direction for usability of the design for the quality report, they provided less so for the content of the quality report.

Third, to gain a better understanding and guide decision-making which 82304‐2 quality requirements would benefit from detailed information, the HCP Advisory Board proposed and AM organized a web-based survey (PD technique: stakeholder survey). The survey—in English only—aimed to evaluate which 82304‐2 quality requirements are considered most useful or, put otherwise, have most “use value” [43] in HCP decision-making on recommending a health app. PD methods intend to facilitate a design process in which *values*, in this case related to HCP information needs, emerge and become inscribed in a prototype, aiming for a final prototype (ie, design) that fits its intended future users’ values [30]. Medical societies assisted in recruiting a convenience sample of HCPs via email. No further inclusion or exclusion criteria were applied. Thirty-one HCPs participated. They were asked to provide demographics (age, gender, and country of origin), individually tick in an Excel sheet only those 82304‐2 quality requirements that they believed provided information valuable for HCPs in their decision-making on recommending a health app, and return their responses within 2 weeks. The survey was not piloted. Descriptive statistics (mean, SD, percentages) were used to summarize the results and identify the 82304‐2 quality requirements considered relevant by at least 50% of the respondents.

Fourth, to generate new ideas and arrive at the 82304‐2 quality report design, participatory prototyping, one of the most important methods used in PD [30], was applied (PD technique: prototyping with stakeholders). In participatory prototyping, participant values are translated into design requirements (here referred to as key design decisions to avoid confusion with the 82304‐2 quality requirements) [28], which iteratively materialize in what is intended to be a high-fidelity prototype (ie, design) as a result of interactions between designers, co-designers, and material objects (mock-ups, prototypes, etc) [30]. Prototyping has been found to support PD principles “mutual learning” and “tacit or latent needs assessment” [44]. PH, designer and researcher of the 82304‐2 quality label, trained and experienced as an industrial engineer and change manager, based in the Netherlands, translated the participant values, key design decisions that emerged, and own handwritten notes from the HCP Advisory Board meetings in iterative 82304‐2 quality report prototypes. Postmeeting, premeeting, and prototyping reflections by PH and AM, medical professional based in Lithuania, past–vice president of the European Junior Doctors Association, an organization for which digital health, including training, pan-European standards, guidelines, and research, is a key pillar, helped shape the iterative prototypes and determined timing and agenda for the next HCP Advisor Board meetings. This researcher role can be understood as that of a main participant directly participating as a designer in the design of a product [45]. PH consulted when needed lead app assessors from the Organisation for the Review of Care and Health Apps (ORCHA) based in England to ensure new iterations of the quality report prototypes could realistically be produced in the future. PH initiated and maintained a Word document which specified for each of the 82304‐2 quality requirements what information would be included in the quality report if the requirement was met, not met, or not applicable. This content was informed by parallel project tasks which resulted in a handbook app assessment for 82304‐2 aligned with EU-level legislation. PH kept Oscar Smeulders, graphic designer of the 82304‐2 quality label, also based in the Netherlands, informed about updates in the Word document. Oscar Smeulders proposed and used as a material object a FIGMA graphical design software visualization for the graphical design of the 82304‐2 quality report.

##### Final Feedback Meetings

Three separate final feedback meetings were organized to enable all 9 members of the HCP Advisory Board to provide their final remarks. Another 2 separate final feedback meetings were organized to gather feedback from in total 8 medical societies. In these final feedback meetings, PH and AM presented the final 82304‐2 quality report design prototype (the FIGMA visualization) and the detailed information in it (the Word document). Questions from the participants were answered, and feedback captured in notes by PH, AM, and PAP. PAP compiled and presented at the end of these meetings the web-based questions (PD technique: stakeholder survey) which were used to assess readiness of the final design prototype for evaluation in a future effectiveness study (“design saturation”) [28]. These questions also indicatively assessed the quality report’s potential usefulness for medical societies in providing guidance on recommending health apps. See Multimedia Appendix 2 for the web-based questions, which were inspired by the Theoretical Domains Framework (TDF), previously employed by Biliunaite et al [26] to investigate barriers to HCP health app recommendation behavior. Horizontally stacked bar charts were used to summarize the results. Responses to a final open question and the feedback in the meetings informed the final design of the 82304‐2 quality report. This final version of the health app quality report was shared with 2 employees with a focus on digital health of the Dutch Patient Federation to gather their feedback on usefulness and usability of the quality report for patients. To support their adequate understanding and enable valuable feedback, all their written questions were answered in writing in 2 consecutive rounds.

##### Comparative Content Analysis and HEMAT

A comparative content analysis and the 8-item HEMAT were used to evaluate the content and understandability of 4 health app quality reports (PD technique: competitive benchmarking). HEMAT was created by the United States National Library of Medicine and is based on the understandability items from the Patient Education Materials Assessment Tool for Printable Materials developed by the United States Agency for Healthcare Research and Quality [46]. Heatmaps were used to visualize the results. Rationale for the focus on content and understandability derives from the identified need for more well-founded detailed information than the DiGA directory [17,18] and a brief uniform understandable explanation within a few clicks [27]. The 4 quality reports included (1) the final FIGMA design of the 82304‐2 quality report, (2) the German DiGA directory [12], (3) the Finnish Digi-HTA directory [35], and (4) AppGuide, a private Canadian initiative [37]. For the comparative content analysis, PH and AM examined the type of detailed information available and the number of clicks required to access the detailed information. PH, AM, and an understandability expert from Pharos, the Dutch Center of Expertise on Health Disparities, who is a GP by background, completed the HEMAT (Textbox 1). To assess HEMAT item 2 , the literature on the type of information HCPs need [17,18,22,27] was used. To add nuance, the regular yes/no scoring for HEMAT was enhanced to yes/in part/no.

Textbox 1. **The 8 items of the Health Education Materials Assessment Tool.**

1. An obvious main message near the top to make the purpose of the report evident.
2. All the relevant information readers need to take action, excluding information that may distract or overwhelm users unfamiliar with the topic.
3. Common, everyday language; necessary medical terms are explained if used.
4. For the most part, active voice (eg, x does y instead of y is done by x).
5. Short sections with headings to easily do a quick scan and find needed information.
6. Selective use of visual cues (eg, font size, arrows, boxes, bullets, and highlighting) to draw attention to key takeaways before reading the words.
7. Visual aids (eg, photos, illustrations, and graphics) to support the main message.
8. Numbers are easy to understand (eg, whole numbers instead of decimals, 3 out of 10 instead of 30%, 3rd instead of third, and no need for readers to make calculations).

### Outcome Measures

Outcome measures for PD can range (1) development (eg, ideas developed), (2) quality (eg, understandability), and (3) user outcomes of the quality report (eg, intent to prescribe or actual prescriptions) [33]. In this study, the key design decisions that emerged during participatory prototyping are the development outcome measures. The web-based questions in the final feedback meetings (PD technique: stakeholder survey), the comparative content analysis (PD technique: competitive benchmarking), and the HEMAT (PD technique: competitive benchmarking) are the quality outcome measures. A proposal for an effectiveness study has been submitted to assess user outcomes, results of which, if funded, will be reported separately. The proposed study will include both HCPs that are known to have more affinity with health apps (young urban HCPs) and HCPs that are known to be more reluctant (older rural HCPs) and will use widely applicable clinical situations (“vignettes”) to approximate real-life HCP scenarios [18].

### Ethical Considerations

The study followed a participatory and emergent research approach conducted within the framework of an EU-funded project. Due to the exploratory and iterative nature of the activities, formal ethics committee approval was not obtained prior to the study. However, ethical considerations were integrated throughout the research process and participant rights were actively protected. Participation in the HCP Advisory Board, the prioritization survey, and final feedback meetings was entirely voluntary, and participants could discontinue participation at any stage. Participants in the HCP Advisory Board confirmed their willingness to participate via email communication and were informed at the beginning of meetings about the purpose of the discussions, the intended use of the collected information, and their right to withdraw or decline to contribute without any consequences. For the survey-based data collection, participants were informed about the purpose of the study and provided consent through the Google Forms interface prior to participation. Participants in the feedback meetings were informed that they could leave the meeting at any time if they did not wish their opinions to be included in the research. The employees of the Dutch Patient Federation who provided feedback signed an informed consent form. The study did not involve vulnerable populations, medical interventions, or the collection of highly sensitive personal data. Data collection focused on professional experiences and perspectives relevant to the project activities. All collected information was handled in accordance with applicable data protection and confidentiality principles within the EU project framework. All members of the HCP Advisory Board received and reviewed the final version of this manuscript and agreed to its publication. See Multimedia Appendix 3 for the reporting guideline for health research involving design [47].

## Results

### Participatory Prototyping 82304-2 Quality Report

The HCP Advisory Board met 12 times over the course of 18 months (December 8, 2022, to May 29, 2024). The recommended group size of 4-8 [28] was achieved in the iterative meetings, which lasted in between 1 and 1.5 hours. The survey to evaluate the usefulness of the 82304‐2 requirements in decision-making on recommending a health app revealed that in total 37% (30/81) of 82304‐2 requirements were prioritized by at least 50% of the participating 31 HCPs. The medical societies that were contacted to support recruitment, the demographics of the 31 HCPs, and the 30 prioritized 82304‐2 requirements are included in Multimedia Appendix 4. The most prioritized 82304‐2 quality requirement, endorsed by 87% (27/31) of the participating HCPs, was “Peer-reviewed scientific literature used in the development of the app.” The second most prioritized 82304‐2 quality requirements were “Health issues targeted,” “HCPs involved in the development of the app.” “Potential customers and users made aware of health risks,” “Health benefit evidence,” and “Appropriate evidence level for the health benefit,” which were all selected by 80% (25/31) of the participating HCPs. The key design decisions (“development outcomes”) that emerged in the participative prototyping of the 82304‐2 quality report are shown in Textbox 2.

Textbox 2. **Key design decisions.**

1. If feasible, have the 82304‐2 quality report satisfy both health care professionals’ (HCPs’) and patients’ information needs instead of a separate version of the report for HCPs and another version for patients (“there are very experienced patients and very unexperienced HCPs and it’s good if they speak the same language”).
2. A design that aligns with the 82304‐2 quality label design.
3. Maintaining the 82304‐2 quality assessment framework structure including the scores of “Healthy and safe,” “Easy to use,” “Secure data,” and “Robust build.”
4. Within a few clicks (“everything is important but not all at once”) access to an increasing detail of information.
5. An in one glance overview of the assessment results (requirement met, not met, or not applicable) for the 67 score-impacting 82304‐2 quality requirements using color-coding and abbreviated standardized accessible text.
6. More information detail beyond if the 82304‐2 quality requirement was met, not met, or not applicable where considered value adding based on the prioritization survey and HCP Advisory Board feedback.

### Final Feedback Meetings

The responses from the HCP Advisory Board to the web-based questions inspired by the TDF (Figure 3) revealed that the HCP Advisory Board members were very positive about the 82304‐2 quality report final prototype: 89% (8/9) agreed or strongly agreed that it was well constructed and 100% (9/9) agreed or strongly agreed that it offered all information and materials that HCPs need to recommend high-quality apps to their patients. In total, 100% (9/9) of the HCP Advisory Board members agreed or strongly agreed to medical societies or organizations having a responsibility to produce guidance on recommending high-quality health apps, while 78% (7/9) agreed or strongly agreed to medical societies having a responsibility in providing training on recommending high-quality health apps. Although the 82304‐2 quality report design was not built with this purpose in mind, 56% (5/9) agreed or strongly agreed that the final prototype nonetheless offered all information and materials for medical societies to produce guidance. The Board members confirmed a perceived need for assistance for medical societies in producing guidance: 100% (9/9) agreed or strongly agreed. Relatedly, 56% (5/9) disagreed with medical societies having clear plans to produce guidance on recommending high-quality apps.

**Figure 3.**
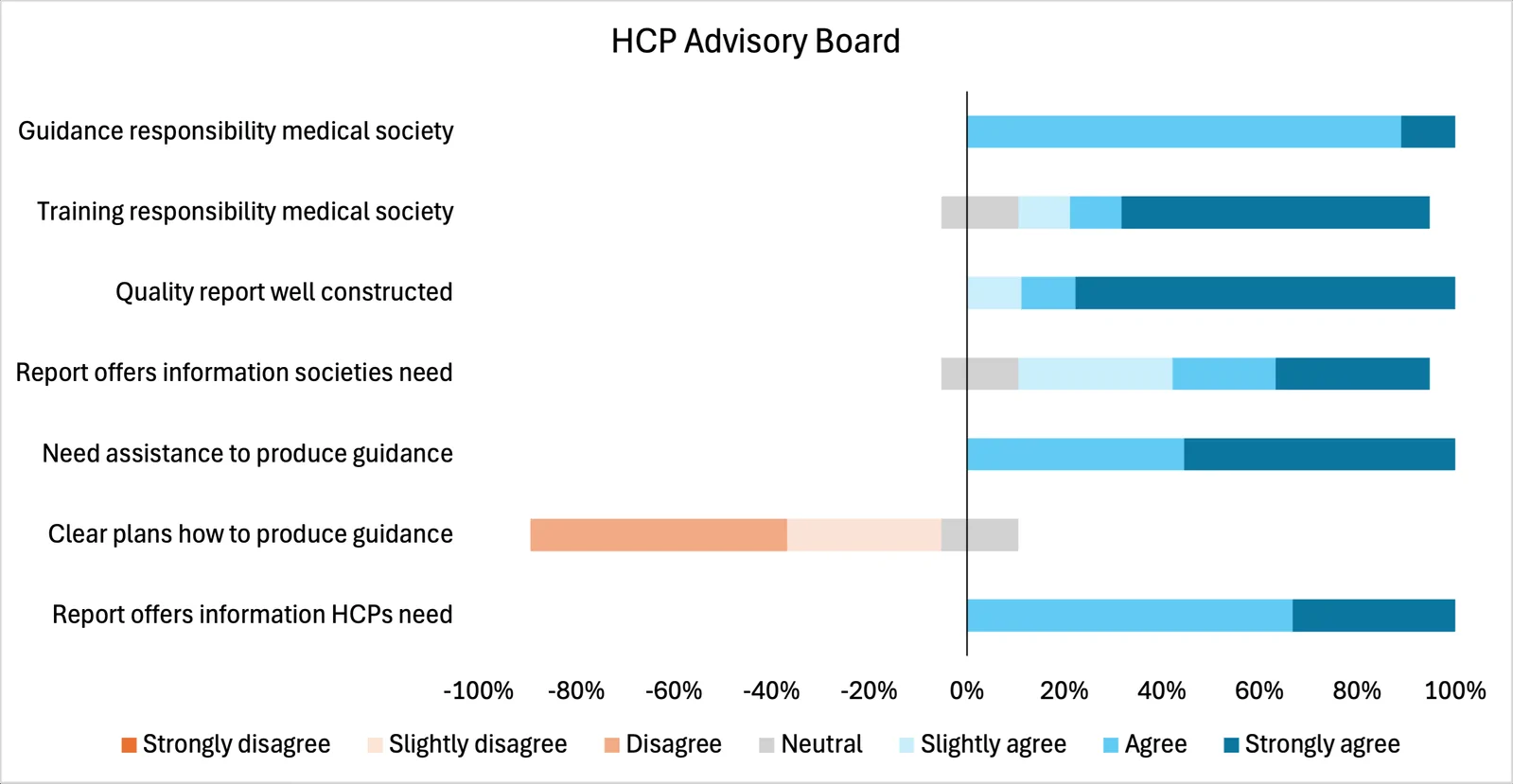
Responses of the 9 HCP Advisory Board members to the 7 questions inspired by the Theoretical Domains Framework. HCP: health care professional.

The responses from the medical societies to the web-based questions inspired by the TDF (Figure 4) revealed that the medical societies were also positive about the 82304‐2 quality report final prototype: 88% (7/8) agreed or strongly agreed that it was well constructed. Although the report was not designed with medical societies in mind, 50% (4/8) agreed or strongly agreed that it offered all information and materials for medical societies to create guidance. The results indicated mixed to positive perceptions regarding medical societies’ responsibility to produce guidance on recommending high-quality health apps: 63% (5/8) agreed or strongly agreed and 25% (2/8) slightly disagreed or disagreed. Responses were more reluctant to negative about providing training: 0% (0/8) agreed or strongly agreed, 38% (3/8) slightly agreed, and 38% (3/8) disagreed or strongly disagreed. The results confirmed that medical societies need assistance in developing guidance on recommending high-quality apps to members: 63% (5/8) agreed or strongly agreed. This need was reflected in the negative responses to having clear plans for producing such guidance: 63% (5/8) disagreed or strongly disagreed.

The final remarks, related follow-up to transform the final prototype into the final 82304‐2 quality report design, and the medical societies that participated in the final feedback meetings are included as Multimedia Appendix 5. Two items related to the concept that “an app is not a pill” require further research. These concern a taxonomy of health interventions or health app functionalities and sufficient level of evidence (accepted study design) for different intended uses.

**Figure 4.**
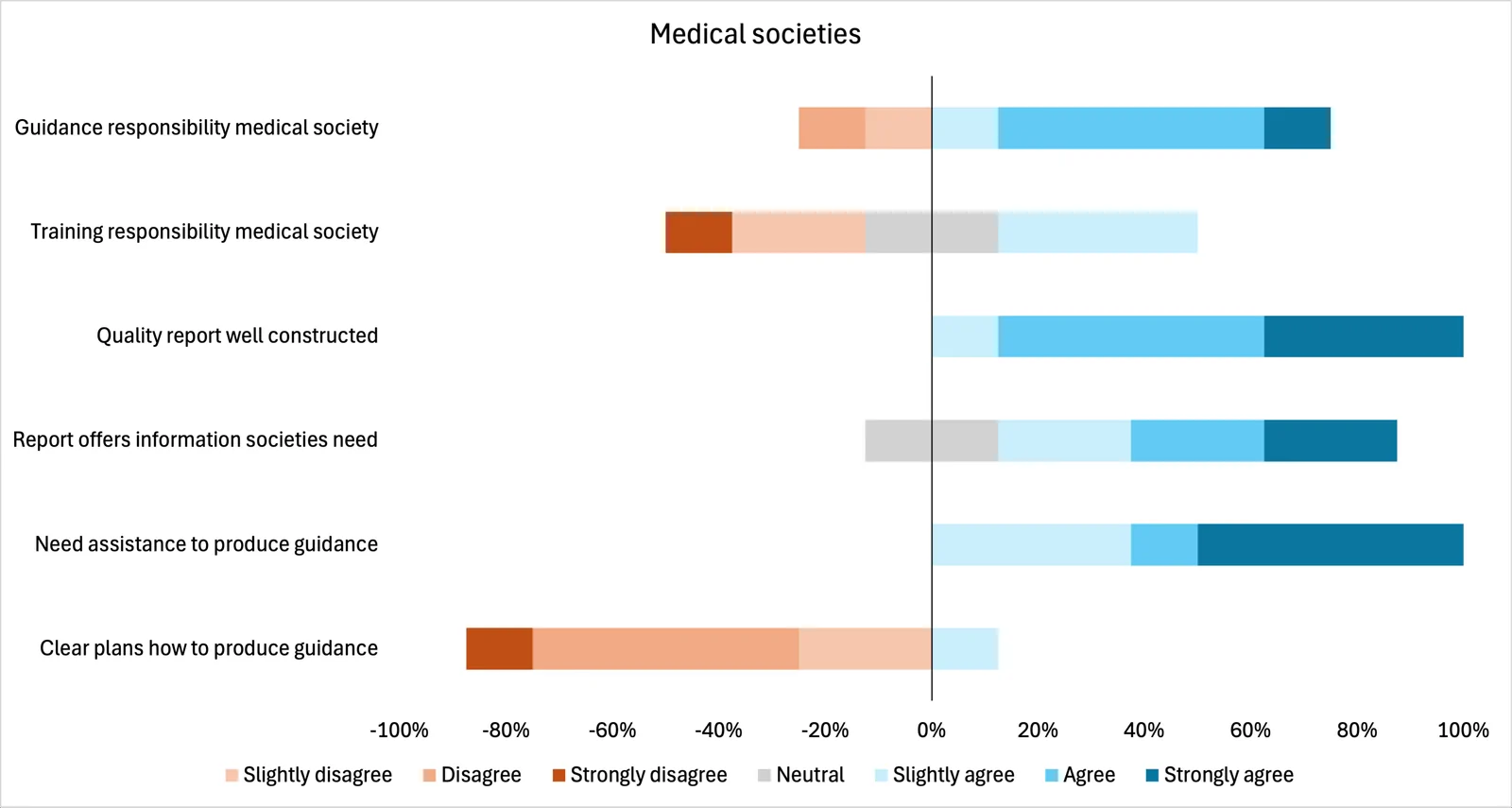
Responses of the 8 representatives of the medical societies to the 6 questions inspired by the Theoretical Domains Framework.

### 82304-2 Quality Report Design

The final FIGMA graphical design software visualization of the design of the 82304‐2 quality report, scrollable and in part clickable to visualize access to the more detailed information, is included in Multimedia Appendix 6. See Figure 5 for screenshots of the FIGMA. The final version of the Word document, which specifies for each of the 82304‐2 quality requirements what information will be included in the 82304‐2 quality report if the requirement is met, not met, or not applicable, is included in Multimedia Appendix 7.

All key design decisions were adhered to (Textbox 3).

**Figure 5.**
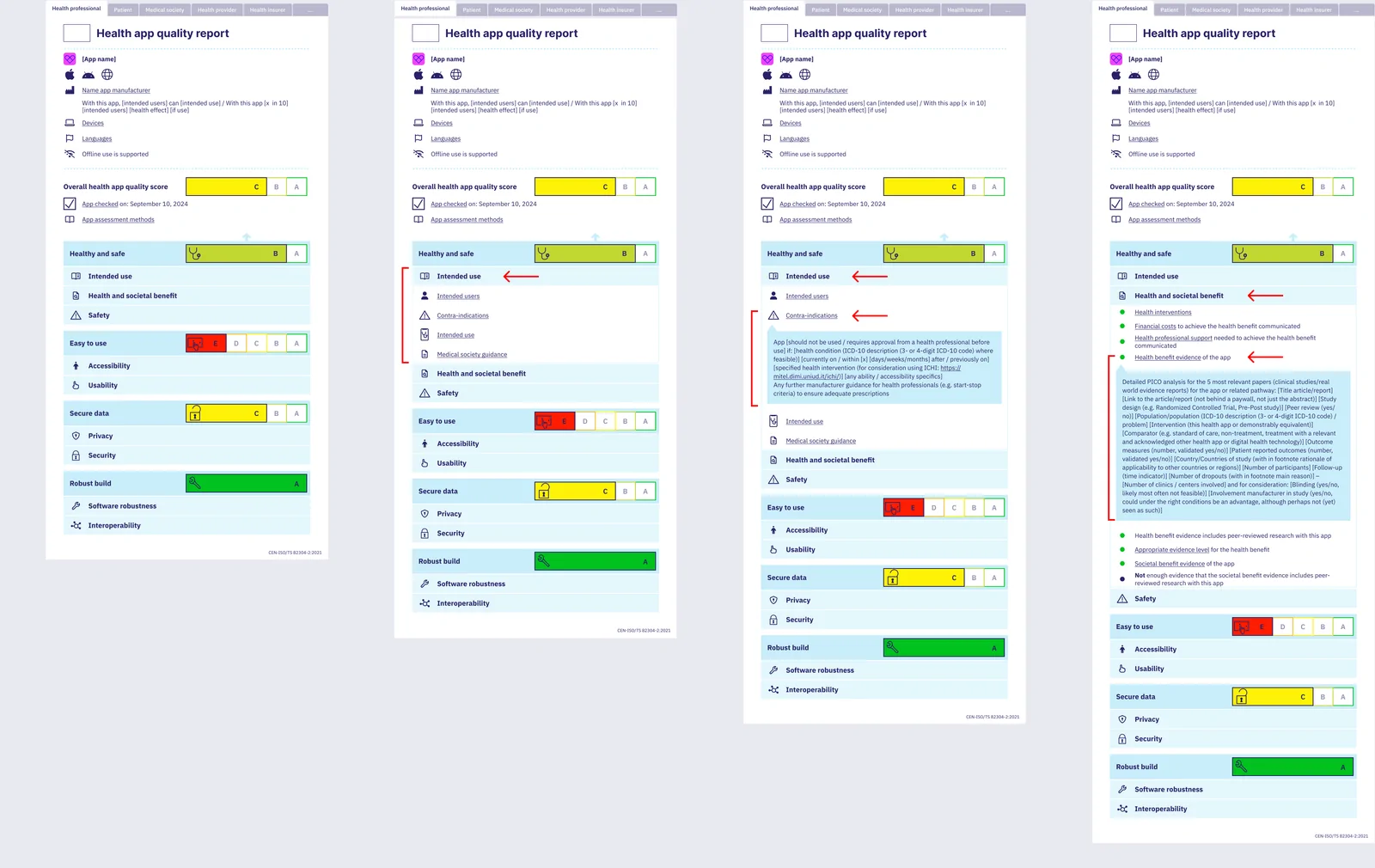
Screenshots of the design of the CEN-ISO/TS 82304‐2 health app quality report (FIGMA graphical design software visualization). The red arrows were added to indicate what has been clicked; in the second screenshot subcategory “Intended use”; in the third screenshot subcategory “Intended use” and the 82304‐2 health app quality requirement regarding “Contra-indications”; and in the fourth screenshot subcategory “Health and societal benefit” and the 82304‐2 requirement regarding “Health benefit evidence.” The red vertical lines were added to indicate what has opened with the last click. The fourth screenshot also displays the colored dots (green/dark blue/grey) that indicate whether an 82304‐2 requirement is met (yes/no/not applicable). Only subcategories (“Intended use,” “Health and societal benefit,” etc) and underlined quality requirements can be clicked to provide access to more detailed information. The square on top is intended to display a flag (eg, the EU flag, similar to the EU energy label) if endorsed by a regional or national authority. The necessity for stakeholder-specific versions of the 82304‐2 quality report and related need for the separate tabs at the top (health professional, patient, etc) is to be established in follow-up research.

Textbox 3. **Adherence to key design decisions.**

1. A consultation of the Label2Enable Patient Advisory Board, established and chaired by the European Patients’ Forum, indicated that patients’ information needs with regard to health app decision-making were quite similar to health care professional (HCP) information needs. To accommodate potential different versions of the quality report targeted at meeting different stakeholder information needs over time, tabs were added at the top of the quality report design. Feedback from the Dutch Patient Federation confirmed the value of the content of the final version of the quality report for patients, and the importance of design decisions such as the use of color-coding and abbreviated standardized text for understandability. Minor changes were made, in particular, the suggestion to explain to patients what to expect from “Medical society guidance.”
2. The quality report design aligns with the 82304‐2 quality label design, using similar colors and fonts.
3. The quality report maintains the 82304‐2 assessment framework structure, including the scores for the quality aspects “Healthy and safe,” “Easy to use,” “Secure data,” and “Robust build.” Details immediately visible in the quality report which are not included in the quality label include which devices are supported (computer, tablet, smartphone, smartwatch, and other), languages in which the app is available, whether offline use is supported, a link to the assessment methods, and subcategories within scores to facilitate focused access. The 5 subcategories within “Healthy and safe” in the 82304‐2 framework were reduced to 3 and phrased “Intended use,” “Health and societal benefit,” and “Safety,” priority categories in the literature, as well as in discussions in the HCP Advisory Board following the use value evaluation. “Technical robustness” was rephrased “Robustness.” Other subcategories remained as is.
4. Detailed information on an 82304‐2 quality requirement level is accessible in 2 clicks.
5. With 1 click on a subcategory, the quality report provides a quick overview of the assessment results of all score-impacting 82304‐2 quality requirements within that subcategory using color-coded dots. A green dot ahead of a quality requirement indicates that the requirement is met, dark blue indicates that the quality requirement is not met, and gray indicates that the quality requirement is not applicable. Standardized short simple texts are used to ensure adequate interpretation of the colored dots. For example, a requirement that is not met starts with **Not** enough evidence, with Not in bold.
6. With a second click on a quality requirement that is met (green dot) and underlined (indicating a hyperlink), the quality report provides access to more detailed concise factual information on that individual quality requirement. Detailed information is available for 90% (27/30) of the 82304‐2 quality requirements that were prioritized by at least 50% of the convenience sample of HCPs. Adding detail for the remaining 10% (3/30) quality requirements, of which 1 had 55% and 2 had 52% endorsement, was carefully considered with the HCP Advisory Board but judged not to add value. Even if this level of detail would not be accessed 99 out of 100 times by an individual HCP deciding on an app, the HCP Advisory Board considered this detailed information vital to be included for the 1 out of 100 times, to avoid a duplication of assessment efforts and to add to trust.

### Comparative Content Analysis

The results of the comparative content analysis are included in Multimedia Appendix 8. Except for AppGuide (4 clicks), all reports provided detailed information in at most 2 clicks. The 82304‐2 quality report provides detailed factual information for more quality requirements than DiGA, Digi-HTA, and AppGuide, and with a few exceptions in equal or fewer clicks. Of note, our design effort did not include a directory that provides access to multiple health app quality reports, which might add a click. DiGA differed in having a separate section for HCPs (“Information für Fachkreise”), in not providing (color-coded) quality scores, and in having products either permanently or provisionally listed or not listed anymore, indicating whether funding by statutory health insurance funds is provided. DiGA did provide search options (medical specialty, platform, ICD [International Classification of Diseases] indication, status of the app [permanently included, provisionally, and removed], age [12‐17 years, 18‐65 years, and older than 65 years], gender, language, additional costs, and additional devices [no, included, optional, and necessary]). As the supply of DiGA grows, choosing between DiGA with the same intended use and intended users may become challenging without (color coded) quality scores [48]. To indicate whether individual health app quality requirements were met, AppGuide and 82304‐2 used color coding and standardized text, DiGA-only standardized text, and Digi-HTA descriptive information.

### HEMAT

The HEMAT understandability results are included in Multimedia Appendix 9. The 82304‐2 quality report scored highest with 63% (5/8) of items scored yes and 25% (2/8) in part, AppGuide scored second with 57% (4/7) yes and 14% (1/7) in part, Digi-HTA scored 13% (1/8) yes and 25% (2/8) in part, and DiGA scored 13% (1/8) yes and 13% (1/8) in part. Of note, DiGA and 82304‐2 scored best on providing all the information HCPs need, with details including indications and contraindications using ICD-10 (International Classification of Diseases, 10th Revision), functionalities, a standardized granular overview of privacy measures met and not met, and a PICO (Patient or Problem, Intervention, Comparison, Outcome) analysis for clinical evidence. However, DiGA did not score on “excluding information that may distract or overwhelm users unfamiliar with the topic.” AppGuide and 82304‐2 both provide access to detailed information per individual requirement, whereas DiGA and Digi-HTA provide this access to findings for a range of quality requirements within a category. Especially for DiGA, this results in long text documents, which makes finding needed information more challenging. Digi-HTA and AppGuide did not provide this information, or not in the needed granularity. Digi-HTA assesses “suitability of an app for use in healthcare” in general and, as such, does not seem to tailor to the information needs of HCPs in their decision-making for individual patients. AppGuide was distinctive in information on all (322.020) health and wellness apps in the app stores, providing HCPs with a source of information to respond to questions from patients who have come across a health app themselves.

## Discussion

### Principal Findings in Perspective

In this study, we used PD techniques to design the 82304‐2 quality report, with an aim to support decision-making on recommending an individual health app to an individual patient by providing HCPs in a user-friendly way the information on the quality of a health app that fits their needs and competences. We managed to engage the Label2Enable HCP Advisory Board, a diverse group of 9 HCPs with expertise in digital health, in participatory prototyping over the course of 18 months, supplemented by 8 medical societies in a final feedback meeting. This resulted in a final 82304‐2 quality report design with detailed information for 90% (27/30) of the 82304‐2 quality requirements HCPs (n=31) perceived of most use value in individual HCP decision-making on recommending a health app. A comparative content analysis and the HEMAT indicated that the quality report design was generally comprehensive and understandable. Final feedback from the 9 HCPs and 8 medical societies was limited, and responses to our web-based questions on the final prototype confirmed promising potential in meeting HCP information needs (“design saturation”). Although the quality report was not designed with the needs of medical societies in creating guidance on recommending high-quality health apps in mind, the quality report design did show potential in also meeting medical societies’ information needs. Feedback from the Dutch Patient Federation indicatively confirmed the usefulness and usability of the quality report for patients. Two items in the quality report related to “an app is not a pill” require further research. These are a taxonomy for health app functional components and, on a more granular intended use level, which study designs are considered adequate evidence.

The recent mapping of HCP information needs and the 82304‐2 quality assessment framework created with the European Society of Cardiology Regulatory Affairs Committee [23] affirms the potential of 82304‐2 to satisfy HCP and potentially also medical society information needs with regard to recommending health apps. More conclusive evaluation of the usefulness and usability of 82304‐2 quality reports by HCPs is recommended, also considering guidance that formal evaluations such as testing comprehension, usability, behavioral intention, or behavior change should follow PD’s preliminary evidence of meeting objectives [28], and the few interventional studies to enhance HCP health app recommendations to date and their limitations [21]. These evaluations should make use of 82304‐2 quality reports with the results of actual app assessments instead of dummy content. The assessed apps should have target users that fit the patient characteristics of the participating HCPs. A particular focus on reluctant and less experienced HCPs should be considered to ensure that the quality report effectively meets the information needs of both HCPs who have and those who have not yet been willing and able to recommend health apps. Both German and French research suggest 3 types of HCPs, being those with positive attitudes toward health apps based on perceived benefits (eg, facilitating and complementing care provision and behavioral effects for patients), those carefully considering benefits (eg, effectiveness and efficiency) and risks (eg, overuse and misuse) for patients and HCPs, and those with negative attitudes (eg, concerns about data privacy, liabilities, actionability of app data, and adverse effects on patient-doctor relationship) [3,16]. Additionally, further research efforts with medical societies and other stakeholders will have to prove the multistakeholder and intercontinental value of the 82304‐2 quality report and related potential in avoiding a duplication of assessment efforts. Such evaluations are expected to enhance the compatibility of the report with the needs of a wide range of HCPs, their medical societies, and with extended efforts potentially more stakeholders that matter to HCPs, such as patients, hospitals, insurers, and HTA bodies. Nontraditional stakeholders (eg, new market entrants such as a telecom company or supermarket that recommends prevention-related apps) may also be considered [49-51].

Medical societies were more ambivalent to negative about their role in providing guidance and training on recommending high-quality health apps than we expected based on medical societies’ common roles in guidance and training and the literature on HCP needs as to medical society guidance with regard to recommending health apps. The perceived need of medical societies for more assistance in creating this type of guidance seems to indicate the lack of clear plans, and perhaps also the ambivalence may be more related to a lack of know-how than a lack of interest. The particularly negative score for training may be due to difficulties designing a training if guidance lacks. Further research and support for medical societies to develop effective guidance and related training seem warranted, ideally without each and every medical society having to invent or reinvent the wheel. We expect that an effort with a limited number of medical societies will reveal that guidance will be only to a limited extent health issue specific. This finding would prove directional to further medical societies and provide a basis for creating guidance and training material (eg, Massive Open Online Courses) for both the medical societies involved and their not directly involved peers. Potential new medical societies such as the German Medical Society of Digital Medicine [52] can be involved in the future—in addition to more industry-guided societies of health app manufacturers.

This study was part of the Horizon Europe Label2Enable project. Another task in this project examined the EU label legislations for energy, food, and medication. All these labels were found to be accompanied by more detailed quality reports. These reports were made available in a database, as were the 3 quality reports we compared with in this study. Although there are many health and wellness apps, their numbers are limited compared with, for example, the number of household appliances in the European Product Registry for Energy Labelling and medical devices in the European Database on Medical Devices. In this research, we created a design for the 82304‐2 quality report. A working scalable version of the 82304‐2 quality report, also referred to as “proof of process” and “proof of production” [44], as well as the related open access database, requires additional funding and yet could build on the learnings with the energy, food, and medication labels, and perhaps even on their existing (EU) platforms or the yet to be established EU database for registration of electronic health record systems and wellness applications described in Article 49 of the European Health Data Space regulation. Having the designated platform connect to routinely used trusted platforms, such as those of medical societies, may be further explored, benefiting from experiences in, for instance, Australia [7], and findings in research in Germany [16,18], where, for example, 73% of diabetes HCPs obtained information on apps from their medical society versus 13% from the DiGA directory and 7% from the national health portal [16].

### Strengths and Limitations

To our knowledge, this is the first extensive PD study to arrive at a design for a health app quality report with indicative proof of satisfying the information needs of HCPs and potentially other stakeholders, in particular, medical societies and patients. The perceived potential alongside complementary interventions toward making recommending high-quality apps a habit and building HCP confidence in this new skill [26] holds promise in addressing the current lack of high-quality health app recommendations and achieving the related health and societal benefit. Although 82304‐2 is a standard with global applicability, and the 81 global quality requirements were the basis of the 82304‐2 quality report, the information provided including granularity may be specific to information needs of European HCPs. In total, 89% (8/9) of HCPs and all medical and patient societies involved were European. Some of the detailed information in the quality report was adapted to the EU regulatory context (eg, referring to the EU Medical Device Regulation and the [soon to be implemented] European electronic health record exchange format), as was some of the wording (eg, personal data instead of personally identifiable information, and data protection officer instead of person responsible for legal and regulatory compliance of processing of personally identifiable information, following terminology used in the EU General Data Protection Regulation). Differences may also be attributable to health system specifics and context-specific additional quality requirements [53]. We acknowledge that in the aftermath of the COVID-19 pandemic the number of HCPs who evaluated the use value of individual 82304‐2 requirements (n=31) as well as the number of participants in the final feedback meetings (n=17) was limited, and that the HCP Advisory Board consisted largely of HCPs with experience in the field and interest in the topic, who were also involved in the participatory prototyping. This likely had an effect on the responses to our questions based on the TDF with regard to the quality report being well constructed and offering the information that HCPs need. On the other hand, the 8 medical societies that participated in the final feedback meetings were not involved in this design effort and agreed with the quality report being well constructed. We recognize that our current work concerned prototyping and not real-life deployment. We recommend more conclusive evaluation of usefulness, usability, and use of the 82304‐2 quality report by HCPs in a demonstrator phase or effectiveness study, with particular effort to also include reluctant and less experienced HCPs and other relevant stakeholders. This phase would also enable next stages of the design process, progressing from “proof of concept” and “proof of product” to “proof of process” and “proof of production” [44], beyond the current indicative assessments of ORCHA lead app assessors as to feasibility to produce and scale production of the 82304‐2 quality report.

### Conclusions

We succeeded in our aim to design the 82304‐2 quality report including its information detail and found promising potential for its distinctive usefulness for HCPs in recommending health apps, complementing the 82304‐2 quality label for which an effect on willingness to recommend apps was previously found. Additionally, initial feedback on the potential compatibility of the quality report for medical societies in creating guidance and for patients in considering a health app was positive. Further efforts are needed to test and fine-tune the HCP, multistakeholder and intercontinental usefulness, usability, and use of the 82304‐2 quality report; to support medical societies in providing guidance and training; and to evolve from the current FIGMA design to a working scalable version of the 82304‐2 quality report and related open access database.

## Supplementary material

10.2196/86473 Multimedia Appendix 1 HCP Advisory Board.

10.2196/86473 Multimedia Appendix 2 Questions final feedback meeting.

10.2196/86473 Multimedia Appendix 3 Reporting guideline for health research involving design.

10.2196/86473 Multimedia Appendix 4 Priority 82304-2 requirements survey.

10.2196/86473 Multimedia Appendix 5 Follow-up final feedback.

10.2196/86473 Multimedia Appendix 6 82304-2 quality report design (“FIGMA”).

10.2196/86473 Multimedia Appendix 7 82304-2 quality report information detail (“Word document”).

10.2196/86473 Multimedia Appendix 8 Comparative content analysis.

10.2196/86473 Multimedia Appendix 9 Health Education Materials Assessment Tool results.

## Abbreviations

82304-2: CEN-ISO/TS 82304-2
CEN: European Committee for Standardization
DiGA: Digitale Gesundheitsanwendungen (Digital Health Applications)
EHR: electronic health record
EMA: European Medicines Agency
EU: European Union
FIGMA: graphical design software
GP: general practitioner
HCP: health care professional
HEMAT: Health Education Materials Assessment Tool
HTA: Health Technology Assessment
ICD: International Classification of Diseases
ICD-10: International Classification of Diseases, 10th Revision
ORCHA: Organisation for the Review of Care and Health Apps
PD: participatory design
PICO: Patient or Problem, Intervention, Comparison, Outcome
SmPC: Summary of Product Characteristics
TDF: Theoretical Domains Framework
