# Supplementary material for: Developing a Health App Quality Report for CEN-ISO/TS 82304-2 to Satisfy Health Care Professionals’ Information Needs on the Quality of Health Apps: Participatory Design Study
Source: J Particip Med. 2026 Oct 23;18:e86473. doi: 10.2196/86473 (PMC13693862; doi:10.2196/86473)
Supplement: Supplementary file 1 [file jopm-v18-e86473-s001.pdf]

Multimedia Appendix 1. HCP Advisory Board

HCP Advisory Board

- Lead: Antanas Montvila (head of innovation at the Hospital of Lithuanian University of Health Sciences Kauno Klinikos, radiologist at the Republican Hospital of Vilnius University, past-vice president of European Junior Doctors Association, Lithuania)
- Secretary: Paulius Povilonis (resident doctor in medical oncology at the Hospital of Lithuanian University of Health Sciences Kauno Klinikos, Lithuania)
- Michael Cardinal (co-founder and CEO of TherAppX, lecturer at the Public Health School of the University of Montreal on digital health accreditation frameworks and patient-centric implementation of digital health tools in clinical practice, pharmacist, Canada), also involved as app assessment organization in the Label2Enable project
- Prof. Fabrice Denis (president of the Institute for Smarthealth (INeS), associate professor of e-health at the Medicine School of Paris Cité, radiation oncologist, France).
- Olga Gershuni (Nursing and Mental Health program director at Health Tech Without Borders, researcher at Maastricht University, nurse, Netherlands)
- Prof. Sebastian Kuhn (professor of digital medicine and director of the Institute for Digitalisation in Medicine at the University Hospital Giessen-Marburg and Philipps University of Marburg, rapporteur for the Standing Committee of European Doctors (CPME) on digital competencies[a], trauma surgeon and orthopedist, Germany)
- Prof. Henrique Martins (professor of digital health, leadership and management at a medical school and 2 business schools, internist, Portugal)
- Dr. Lina Mosch (research fellow at the Institute of Medical Informatics, anesthesiology resident at Charité, Germany)
- Dr. Vitalii Poberezhets (assistant professor at the department of propedeutics of internal medicine in the National Pirogov Memorial Medical University, chair of the e-Health/m-Health group at the European Respiratory Society, pulmonologist, Ukraine)
- Prof. Peeter Ross (professor of e-health in eMedLab at Tallinn University of Technology (TalTech) and radiologist at East Tallinn Central Hospital, Estonia)
- Dr. Stephan Schug (past member of EC eHealth Stakeholder Group, secretary to the joint eHealth working group of the Federal Health Ministry, public health physician, Germany)

[a] https://www.cpme.eu/policies-and-projects/digital-health/digital-competencies
