# Supplementary material for: Developing a Health App Quality Report for CEN-ISO/TS 82304-2 to Satisfy Health Care Professionals’ Information Needs on the Quality of Health Apps: Participatory Design Study
Source: J Particip Med. 2026 Oct 23;18:e86473. doi: 10.2196/86473 (PMC13693862; doi:10.2196/86473)
Supplement: Supplementary file 2 [file jopm-v18-e86473-s002.pdf]

Multimedia Appendix 2. Questions Final Feedback Meeting

Questions HCP Advisory Board	Questions medical societies
1. I think that medical societies / organizations have a responsibility to produce guidance on recommending high-quality apps to their members	1. I think that as a medical society / organization, it is our responsibility to produce guidance on recommending high-quality health apps to our members
2. I think that medical societies / organizations have a responsibility to provide a training on recommending high-quality apps to their members	2. I think that as a medical society / organization it is our responsibility to provide a training on recommending high-quality apps to our members
3. The quality report for health apps is well constructed	3. The quality report for health apps is well constructed
4. The quality report for health apps offers all information and materials that medical societies / organizations might need to produce guidance on recommending high-quality apps to their members	4. The quality report for health apps offers all information and materials that we need to be able to produce guidance on recommending high-quality apps to our members
5. I think medical societies / organizations need to have (more) assistance to be able to produce guidance on recommending high-quality apps to their members	5. I think we need to have (more) assistance to be able to produce guidance on recommending high-quality apps to our members
6. I think medical societies / organizations have clear plans on how to produce guidance on recommending high-quality apps to their members	6. I think we have clear plans on how to produce guidance on recommending high-quality apps to our members
7. The quality report for health apps offers all information and materials that (we) medical professionals need to recommend high-quality health apps to our patients	
8. What further things would you recommend us to do to ensure that ISO/TS 82304-2 and its assessments, label and report provide added value to your organization?	7. What further things would you recommend us to do to ensure that ISO/TS 82304-2 and its assessments, label and report provide added value to your organization?

HCP = health care professional
