# Supplementary material for: Developing a Health App Quality Report for CEN-ISO/TS 82304-2 to Satisfy Health Care Professionals’ Information Needs on the Quality of Health Apps: Participatory Design Study
Source: J Particip Med. 2026 Oct 23;18:e86473. doi: 10.2196/86473 (PMC13693862; doi:10.2196/86473)
Supplement: Supplementary file 3 [file jopm-v18-e86473-s003.pdf]

Multimedia Appendix 3. Reporting Guideline for Health Research Involving Design (REDR)

Item	Paper section	Succinct description	Detailed description	Page number
1.	Title	Title should indicate that the study included a design approach.	Explicit mention of design in the title, what was designed, what process was used, intended outcomes or potential contribution.	1 ("Participatory **Design** Study")
1.1	Abstract	Abstract summarizes the salient components, including background, statement of the problem, approach/ methods, findings, results and conclusion.	Statement of the health problem/design brief applied, noting whether qualitative or mixed method research (or other) was included. Abstract should contain relevant information on the health issue, design practice, methods, results and conclusions.	1-2
2.	Introduction/ background	Overview of the background to the topic, what has been done in the area already and rationale for using the design.	Providing relevant references to the scope of the health issue, any previous work in this area or other ways of addressing the health topic; introduction of the design rationale.	2-5
2.1	Available knowledge	Summary of what is known about the topic and any gaps	Literature review of past studies, knowledge and projects relevant to the problem. Statement of what is known about the problem and what has been done to address it in the past, state of the art.	2-5
2.2	Rationale for design approach	Explanation of design as appropriate to address the topic.	Rationale should be clearly articulated.	5
2.3	Description of design challenge for health	Description of the main research question or health problem that the design-based work aimed to address.	Description of design and health issue, scoping of the challenge and scale or timing at which the design was included (which may result in reframing of the initial research question).	5
2.4	Research aims	Overall aims and objectives of the project.	Explanation of the overall aims and the specific objectives of this work.	5
3.	Methods/ approach	Description of approach used for the research.	Description of research, including design approach used and overall role of design in the work.	5-10
3.1	Theory	Underlying theories that informed this work (if any).	Description of any underlying, conceptual or motivating theories or frameworks used.	5-6
3.2	Process and timeline	Processes or steps and timeline for the research.	Detailed description of the process or processes for applying design and research, full timeline of activities from beginning to end.	6-9

3.3	Research team characteristics and reflexivity	Individuals involved in the research team and characteristics of these. Ways that reflexivity was addressed.	Description of research team and how the team addressed reflexivity.<br>- Geographical origin.<br>- Discipline expertise in health, design and/or complementary disciplines and training.<br>- Rationale for team composition.<br>- Level of proximity to or familiarity with community/population of interest.<br>- Prior relationship with community, wider team and/or other researchers (eg, consultants and implementing staff).<br>- Assumptions/presuppositions regarding health topic and design.<br>- Team attributes that could impact power and participation with community (eg, race/ethnicity, socioeconomic status and gender).	8, Multimedia Appendix 1
3.4	Site selection	Description of study sites selected.	Study setting information, including health background, geographical location, rationale and method for choosing site, and previous use of design, if any, in the setting.	6
3.5	Participant selection and engagement	Process to select participants, description of the participants and explanation of how they were involved in design.	Detailed description of participants in the design process and how the research question or intent led to inclusion; explanation of who the participants were, how they were chosen and contacted, what role they had (eg, interviewees, testers), length of involvement, extent of involvement (ie, codesigned vs consulted only), representativeness or generalizability of the participants, any sampling techniques if used or assessment of completeness of the participant group for design.	6, Multimedia Appendix 1
3.6	Ethical considerations	Ethical precautions taken to protect participants, communities, and personal information.	Ethics and information related to human subjects’ protection for social–behavioral research; documentation of ethics approval (if any) by a review board and participant consent, or explanation for lack thereof; addressing privacy, confidentiality and data security and internationally recognized concepts and guidelines (eg, Declaration of Helsinki and Belmont Report).	10

3.7	Language	Description of any language considerations.	Language in which the project was conducted; if in translation, credentials of translators; checking for accuracy of materials or workshop translation; familiarity of designers and participants with language and cultural nuances.	6, 7 (English only)
3.8	Techniques to understand (data collection tools and instruments)	Techniques or tools used during the research process.	Description of the types of the techniques or tools employed, rationale for method, type of data collected and any changes to methods during data collection. Description of who used research tools and with whom; should be stated separately for phases of discovery or insight gathering and testing or prototyping; what was done with this information and for what purpose it was done; any references to precedents for use.	6-10
3.9	Documentation	Documentation of work undertaken in research.	Statement of how documentation was carried out (eg, notes, videos, and photos), by whom, what specifically was documented (eg, interviews, affinity exercises, and observations) and role of participants in documentation. Types of data collected (eg, potentially listing and numbering of interviews, photos, videos, notes, insight statements, prototypes and pile/card sorts).	8
3.10	Techniques to synthesize	Description of techniques used to synthesize insights, iterate, and analyze data.	Explanation of process for team synthesis, analysis of collected information from the design work (eg, brainstorming and journey maps); inclusion of any data analysis software or use of large data sets to validate insights; description of team members involved in this process.	8
3.11	Validation approaches	Process of checking that insights, prototypes or other products were validated.	Description of additional steps used to verify, validate, triangulate or test the themes emerging (eg, comparing to existing literature, expert review and feedback from participants); summary of process of reflection for researchers to understand their own role in the development of the final products and steps taken to enhance validity of the solution (other than direct testing with a small number of users).	8-9, Multimedia Appendix 5, 8, 9

4.	Results/findings from design research and activities	Summary of findings from design activities, resulting insights, what was designed, what resulted from the work and (if available) impact of activities; report on any secondary or ancillary results.	Description of what was created as a result of the work: intervention, solution, policy, technology, behavior change, service or other result; inclusion of salient features of the solution; noting how these addressed underlying health topic and what happened after design (eg, intervention or product status); documentation of ownership of what was created in the process; description of research prototypes.	10-16
4.1	Design research phase	Summary of major insights or reflection from design activities.	Presentation of design research:<br>- Problem framing.<br>- Design insights.<br>- Development and refinement of insights (diverge/converge).<br>- Description of prototyping and use of measurement/assessment and iterative refinement.<br>- Deliverables (eg, service blueprints, maps, prototypes, storyboard).	10-16, Multimedia Appendix 6, 7
4.2	Decision points	Description of decisions made during the design process.	Key decision-making points and any criteria used as the design process was under way for changes made and iterations.	11, 13-14
4.3	Evidence of change or impact	Summary of any evidence of change or impact to health or other facets of the situation.	Description of change or impact if any was noted, including any negative or null results; if evaluation has been done, what, if any, direct health benefits or other benefits, including social transformation, were identified and description by whom these were identified.	Separate follow-up study described on page 10
5.	Discussion	Reflection on design as an approach to the health topic and the strengths and limitations of the work.	Brief reiteration of findings or results; description of any limitations, strengths or challenges faced during research; description of how the results fit with other solutions for this health topic and how conclusions were arrived at in similar or different ways.	16, 18-19
5.1	Design	Reflection of the application of design to this research topic.	Discussion of the transferability and specific contribution of design; description of how the solution developed is different from previous ones and fills any gap in knowledge; possible interpretation or development of a new model or methodology.	16-18

6.	Conclusion	Implications of this work for the larger field and next steps.	Explanation of the next key research or action to address the health topic and broader fields, noting implications for other practitioners or relevance to other contexts, commenting on the future of design for this domain.	19
7.	Other	Acknowledgements of support, assistance, funding, statement on conflict of interest and authors' contributorship.	- Description of any material interest of those involved in the research, including paid consulting.<br>- Statement of funding (if provided and by whom).<br>- Description of the research team's ongoing commitment to addressing any issues raised by participants during the research.<br>Contributorship<br>- Description statement regarding roles each author had in research and reporting.	19
7.1	Glossary	Definition of key terms	Any terminology, especially design terminology, which may not be understood in the lay, health, or biomedical context and vice versa.	20
