# Supplementary material for: Developing a Health App Quality Report for CEN-ISO/TS 82304-2 to Satisfy Health Care Professionals’ Information Needs on the Quality of Health Apps: Participatory Design Study
Source: J Particip Med. 2026 Oct 23;18:e86473. doi: 10.2196/86473 (PMC13693862; doi:10.2196/86473)
Supplement: Supplementary file 4 [file jopm-v18-e86473-s004.pdf]

Multimedia Appendix 4. Priority 82304-2 Requirements Survey

Medical societies that were asked to assist in recruitment of survey participants:

- European Forum of Primary Care (EFPC) – “to make primary care providers able and willing to take responsibility for the health of the population under their care”
- European Junior Doctors Association (EJD) – “safeguards the interests of 300.000 European junior doctors”
- European Medical Students’ Association (EMSA) – “+130 Faculty Member Organizations, 30 countries, +50.000 members”
- European Rural and Isolated Practitioners Association (EURIPA) – “a growing network of rural practitioners and organizations across Europe to address the health and wellbeing needs of rural communities and the professional needs of those serving them across Europe”
- European Young Family Doctors Movement (EYFDM) – “network for early career family doctors of the European regional branch of the World Organization of Family Doctors (WONCA Europe)”
- World Organization of National Colleges, Academies and Academic Associations of General Practitioners/Family Physicians (WONCA Europe) – “the academic and scientific society for general practice / family medicine in Europe, we represent 47 member organizations and more than 90.000 family doctors in Europe”

Demographics of the 31 respondents:

**Age (years)**	
mean (SD)	38 (10.4)
**Gender**	
male	19/31 (61%)
female	12/31 (39%)
**Country of origin**	
Germany	4/31 (13%)
Estonia	3/31 (10%)
France	3/31 (10%)
Lithuania	3/31 (10%)
Ukraine	3/31 (10%)
Belgium	2/31 (6%)
Greece	2/31 (6%)
Netherlands	2/31 (6%)
Spain	2/31 (6%)
Canada	1/31 (3%)
Finland	1/31 (3%)

Norway	1/31 (3%)
Slovenia	1/31 (3%)
Sweden	1/31 (3%)
Turkey	1/31 (3%)
United Kingdom	1/31 (3%)

The priority 82304-2 requirements from top to bottom are included in the following table. The middle column details the percentage of HCPs which considered the 82304-2 requirement a priority. The right column indicates in how many clicks the detailed information on this 82304-2 requirement can be accessed in the (final) 82304-2 quality report design. For 3 lower scoring requirements, “Consent for sharing personal data with third parties” (55%), “Use of advertising is disclosed and can be declined” (52%), and “Developed with software standards, methods and tools” (52%), more detail did not seem to add value. These did not get a color code.

Additionally, information detail for three 82304-2 requirements is addressed in detail via another 82304-2 requirement. Requirement “App is age appropriate” (68%) has in the meantime in the 82304-2 handbook been extended to: “Is the health app appropriate given the intended user diversity?” This change originated from an evaluation of the Australian app assessment framework [1] which emphasizes taking into account Aboriginal and Torres Strait Islander people in the design of an app, literature review and input from a subject matter expert in digital inclusion. Concerning the original age-appropriateness, hesitance to prescribe especially seems to relate to older users [7]. Appropriateness for older users overlaps with general accessibility measures (eg, visual acuity, motor issues and experience with apps which relates to understandability). These items are detailed in the accessibility statement which is made available. Lastly, a public version of the Data Security Policy is made available. This is expected to address “Security incident response procedure” (52%), and “Security app is tested regularly and at major changes” (52%).

= no detail provided | = detail in 0 clicks | = detail in 1 click | = detail in 2 clicks | = detail in 2 clicks elsewhere

**Priority 82304-2 requirements**	**%HCPs that prioritized requirement**	**No. of clicks for more detail**
5.2.1.7 Is appropriate peer reviewed scientific literature used in the development of the health app?	87%	
5.2.1.3 For which health issue(s) and/or health need(s) is the health app intended?	80%	
5.2.1.6 Are health professionals involved in the development of the health app?	80%	

5.2.2.5 Are potential customers and users of the health app made aware of the health risks, contra-indications and limitations of use?	80%	
5.2.4.5 Is evidence available to support the health benefit of using the app?	80%	
5.2.4.5.2 Is the level of the evidence appropriate?	80%	
5.1.1.2 What is the name of the health app?	74%	
5.1.1.4 In which languages is the health app available?	74%	
5.2.1.1 Who are the intended users of the health app?	74%	
5.2.1.4 What is the intended use of the health app?	74%	
5.2.4.1 Describe the health benefit of using the app.	74%	
5.2.4.5.1 Does this evidence include peer reviewed research involving the use of this health app?	71%	
5.1.1.1 Which operating systems or platforms does the health app support?	68%	
5.2.2.1 Are the health risks of the health app analyzed?	68%	
5.2.3.2 Is the health app approved by an independent ethics advisor or ethics advisory board?	68%	
5.3.1.2 Is the health app age-appropriate?	68%	
5.2.2.3 Are the residual risks of using the health app found to be acceptable?	61%	
5.2.2.6 Is a process to collect and review safety concerns and incidents for the health app maintained?	55%	
5.4.1.1.6 Is opt-in the default setting for sharing PII with third parties?	55%	
5.2.1.2 Are age restrictions of the intended users or subjects of care made clear to potential customers and users?	55%	

5.2.2.4 Describe when the health app requires approval from a health professional before use.	52%	
5.2.4.8 Is the use of advertising mechanisms in the health app disclosed to potential customers and users and are these advertisements clearly distinguishable?	52%	
5.3.1.1.3 Are WCAG 2.1 AA compliant measures taken to ensure that all intended users can understand all relevant information and user interface components of the health app and related documents?	52%	
5.3.2.7 Are appropriate resources available to adequately help users who experience problems with the health app?	52%	
5.4.1.1 Does the health app process PII?	52%	
5.4.1.1.4 Is a privacy statement readily available to potential customers and users of the health app?	52%	
5.4.1.1.8 Are security-incident response procedures in place that include reporting PII breaches to the user and relevant authorities?	52%	
5.4.2.9 Are security vulnerabilities reported, identified, assessed, logged, responded to, disclosed, and quickly and effectively resolved?	52%	
5.4.2.10 Are the security of the health app and associated services tested on a regular basis and at major changes?	52%	
5.5.1.2 Is the health app developed with a software development process that covers the standards, methods and tools to be used?	52%	

HCP = health care professional, PII = Personally Identifiable Information

Reference:

1. Assessment framework for mHealth apps. Australian Digital Health Agency.
URL: https://www.digitalhealth.gov.au/about-us/strategies-and-plans/assessment-framework-for-mhealth-apps [Accessed 2026-09-10]
