# Supplementary material for: Developing a Health App Quality Report for CEN-ISO/TS 82304-2 to Satisfy Health Care Professionals’ Information Needs on the Quality of Health Apps: Participatory Design Study
Source: J Particip Med. 2026 Oct 23;18:e86473. doi: 10.2196/86473 (PMC13693862; doi:10.2196/86473)
Supplement: Supplementary file 5 [file jopm-v18-e86473-s005.pdf]

Multimedia Appendix 5. Follow-up Final Feedback

HCP Advisory Board

Final remarks	How addressed in the 82304-2 quality report design
It is hard to make one size fits all principle for different intended uses of the app; different intended use apps should have different assessment schemes	There is a difference for 82304-2 requirement 5.2.4.5.2 “Is the level of the evidence appropriate?” And we do expect that different intended uses will have a different profile of 82304-2 requirements that need to be complied with for medical societies to endorse them, the details of which we hope to find out in an upcoming “Demonstrator phase”
No commercial advertising should be involved	One of the 82304-2 requirements is 5.2.4.8 “Is the use of advertising mechanisms in the health app disclosed to potential customers and users and are these advertisements clearly distinguishable?” In the 82304-2 quality report this is visible as either:<br>● Use of advertising in the app is disclosed and can be declined<br>● **Not** enough evidence that the use of advertising in the app is disclosed and can be declined<br>● No use of advertising in the app<br>To maintain access and keeping in mind health inequities, this likely does require significant more apps being reimbursed than currently, as an app and its maintenance do require another type of (sustainable) funding if it cannot be advertising.

The 8 medical societies that participated in a final feedback meeting were the Standing Committee of European Doctors (CPME), European Junior Doctors Association (EJD), European Medical Students’ Association (EMSA), the European Psychiatric Association (EPA), European Respiratory Society (ERS), European Rural and Isolated Practitioners Association (EURIPA), the European Society for Prevention Research (EUSPR), and the European Regional Branch of the World Organization of Family Doctors (WONCA Europe).

Final remarks	How addressed in the 82304-2 quality report design
**Medical societies**	
Medical app recommendations should be based on clinical guidelines	If available, (EU) clinical guidelines are made available within the Intended use subcategory with 2 clicks
During evaluation intended use should be very clear	Intended use is one of the first questions in “Healthy and safe” and has an impact on the 82304-2 requirements regarding evidence for the health benefit.
Cultural difference regarding the	82304-2 requirement 5.3.1.2 “Is the health app age-appropriate?” has in the 82304-2 handbook been changed to “Is the health app appropriate given the intended user diversity? Cultural diversity has

health app should be addressed	been addressed in the assessment methods with input from a subject matter expert and scientific literature on the topic.
Definition of health interventions should be clearer	The 82304-2 definition of health interventions has been added to the 82304-2 handbook as a note:<br>Note: A health intervention is an act performed for, with or on behalf of a person or a population whose purpose is to assess, improve, maintain, promote or modify health, functioning or health conditions.<br><br>Intent is to make a summary of the handbook available via “App assessment methods” with 2 clicks. We recognize the need for further development of health interventions into a taxonomy. A first draft is:<br>a. Health intervention(s) (for consideration using ICHI: https://mitel.dimi.uniud.it/ichi/)<br>b. Behavior change interventions (e.g. education, training, enablement): https://implementationscience.biomedcentral.com/articles/10.1186/1748-5908-6-42/tables/1)<br>c. Technology-specific interventions (e.g. monitoring symptoms, reminders to promote treatment adherence)<br>d. Algorithms that can [not] change through learning during use with [automated / human] interpretation<br>e. Generative AI (e.g. chatbot)<br>f. Other
It should be clear if the app data can be transferred to electronic health record	The 82304-2 quality report includes (if the health app has application programming interfaces (APIs)) “Interoperability standards used”, “Terminologies used" and “European EHR exchange format is supported”
Security measures and location of data storage of medical app is important	82304-2 includes 11 security requirements, the 82304-2 handbook for these 82304-2 requirements include several “subquestions” that are assessed by the assessor. “Country where data is stored” has been added to the report.
Intervention study with medical apps needed	The level of evidence required for an app within 82304-2 depends on the intended use. For the intended uses “Treat”, “Active monitoring”, “Calculate” and/or “Diagnose” a Randomized Controlled Trial (RCT), systematic review or meta-analysis of RCTs is needed to “pass” quality requirement 5.2.4.5.2 “Is the level of the evidence appropriate?” This is based on the NICE Evidence Standards Framework tiers, which the 82304-2 framework builds on. We recognize the need to make this list more intended use specific, by adding for example for the intended use Diagnose instead of an RCT appropriate evidence precision and accuracy validation and comparative studies to prove clinical utility.

Other remarks - unrelated to the 82304-2 quality report design – were:

- Medical apps: most effort should be put on secondary prevention
- Focus should be put on patient education tools
- Medical organizations should put more effort into addressing medical app guidance demand
- More awareness and education about the 82304-2 quality report needed
- Follow-up work on 82304-2 quality report must be ensured
- Sustainable funding should be sustained for future 82304-2 quality report
- Medical app evaluation should have a legislation on EU level [It does, that is the Medical Device Regulation]
- Medical societies should be a key player in education and recommendation of medical app usage
