# Supplementary material for: Developing a Health App Quality Report for CEN-ISO/TS 82304-2 to Satisfy Health Care Professionals’ Information Needs on the Quality of Health Apps: Participatory Design Study
Source: J Particip Med. 2026 Oct 23;18:e86473. doi: 10.2196/86473 (PMC13693862; doi:10.2196/86473)
Supplement: Supplementary file 6 [file jopm-v18-e86473-s006.pdf]

**Multimedia Appendix 6. 82304-2 Quality Report Design ("FIGMA")**

Access to scrollable and in part clickable FIGMA.
