# Supplementary material for: Developing a Health App Quality Report for CEN-ISO/TS 82304-2 to Satisfy Health Care Professionals’ Information Needs on the Quality of Health Apps: Participatory Design Study
Source: J Particip Med. 2026 Oct 23;18:e86473. doi: 10.2196/86473 (PMC13693862; doi:10.2196/86473)
Supplement: Supplementary file 7 [file jopm-v18-e86473-s007.pdf]

Multimedia Appendix 7. 82304-2 Quality Report Information Detail ("Word Document")

Legend

82304-2 quality aspect:

Quality aspect score:

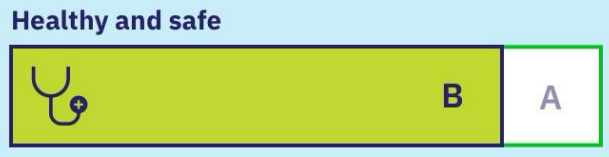

Healthy and safe

Subcategory (clickable to access related 82304-2 quality requirements scores)**:**

Health and societal benefit

82304-2 quality requirement score (color-coding)**:**

- Score impacting question for which evidence was sufficient ("yes")
- Score impacting question for which evidence was not provided or provided but not sufficient ("no")
- Score impacting question was not applicable for this app ("not applicable")

More information available (clickable to access this more detailed factual information): Underlined text

Factual information (to support decision-making, avoid duplication of assessment efforts, in time for potential use in search and selection of apps):
Factual information

82304-2 quality report – information detail for all requirements and response options

[App name]

Name app manufacturer
[Company type] [Registered address] [Telephone number] [Email address]

With this app, [intended users] can [intended use] / With this app [x in 10] [intended users] [health effect] [if use]

Devices
Computer / Tablet / Smartphone / Smartwatch / Other

Languages
[Languages (written in short (to be decided two or three letter code) and in full according to ISO 639)]

Offline use is supported

Not enough evidence that offline use is supported

Overall health app quality score: C | B | A

App checked on [Day Month Year]

[App name] [Version number] was tested in [Language] using [Translation program name] on [Device type] [Version number] in [Platform] [Version number] using [Accessory name] [Version number] against the CEN-ISO/TS 82304-2 assessment guidelines of [Year]

App assessment methods

Link to website healthappqualitylabel.eu/app_assessment_methods for more information on the (rigor of the) assessment, e.g. evidence requested, subquestions, pass / fails, type of assessment (manual / automated), and potentially more information on the certification scheme to e.g. address concerns on conflicts of interest

Healthy and safe

Healthy and safe: B | A

Intended use

Intended users

Anyone / Persons at risk of or diagnosed with [ICD-10 description (3- or 4-digit ICD-10 code and if relevant the additional identifier A, G, V, Z – excluded diagnosis, confirmed diagnosis, suspected diagnosis and (symptom-free) condition after the diagnosis] / Informal carers / Health professionals / Researchers / [Other]
For ages [x] years or younger / [x] to [y] years / [y] years or older
Supervised use for ages [x] years or younger / vulnerable users
[Other to be specified risk minimization measures to optimize the safe and effective use of the health app]

Contra-indications

App [should not be used / requires approval from a health professional before use] if:
[health issue (ICD-10 description (3- or 4-digit ICD-10 code) where feasible)]
[currently on / within [x] [days/weeks/months] after / previously on] [specified health intervention (for consideration using ICHI: https://mitel.dimi.uniud.it/ichi/)]
[any ability / accessibility / health issue severity specifics]
Any further manufacturer guidance for health professionals to ensure adequate prescriptions (e.g. start-stop criteria, restrictions as to type of health professionals to seek approval from)

Intended use

System services / Inform / Simple monitoring / Communicate / Preventative behavior change (Medical Device Regulation: Prevent) / Self-manage (Medical Device Regulation: Alleviate, Compensate) / Research / Treat / Active monitoring (Medical Device Regulation: Monitor) / Calculate (Medical Device Regulation: Predict, Prognose) / Diagnose

Intended use	Description
System services	Health apps that improve health system efficiency. Unlikely to have direct and measurable individual health outcomes. Includes for example electronic prescribing systems, electronic health record platforms and ward management systems [45].
Inform	Health apps that provide information and resources to anyone or persons with, or at risk of, specific health issues. Can include information on specific health issues or about healthy living. Includes for example apps describing a health issue and its treatment, apps providing advice for healthy lifestyles (such as recipes), and apps that signpost to other services [45].
Simple monitoring	Health apps that allow users to record health parameters to create health diaries. This information is not shared with or sent to others. Includes for example health tracking information such as from fitness wearables, symptom or mood diaries [45].
Communicate	Health apps that allow two-way communication between anyone or persons with, or at risk of, specific health issues and health professionals, informal carers, third-party organizations or peers. Health advice is provided by a health professional using the app, not by the app itself. Includes for example instant messaging apps for health and social care, video conference-style consultation software, and platforms for communication with informal carers or health professionals [45].
Preventative behavior change	Health apps that are designed to change intended user behaviour related to, for example, smoking, eating, alcohol, sexual health, sleeping and exercise. Prescribed to intended users by a health professional. Includes for example smoking cessation apps, apps used as part of weight loss programs and apps marketed as aids to good sleep habits [45].
Self-manage	Health apps that aim to help persons with specific health issues to manage their health. Can include symptom tracking function that connects with a health professional. Includes for example apps that allow users to record, and optionally to send data to a health professional to improve management of their health issue [45].
Research	Health apps that generate data for research [56]:<br>— measure the magnitude and distribution of a health problem;<br>— create understanding of the diverse causes or determinants of the problem;<br>— develop solutions or interventions that will help to prevent or mitigate the problem;<br>— implement or deliver solutions through policies and programs; and/or<br>— evaluate the impact of these solutions on the level and distribution of the problem.
Treat	Health apps that provide treatment for a specific health issue (such as CBT for anxiety), or guide treatment decisions. Includes for example apps for treating mental health or other conditions, and health professional-facing apps that advise on treatments in certain situations [45].
Active monitoring	Health apps that automatically record information and transmit the data to a health professional, informal carer or third-party organization, without any input from the user, to inform health management decisions. Includes for example apps linked to devices such as implants, sensors worn on the body or in the home. Data are automatically transmitted through the app for remote monitoring [45].
Calculate	Health apps that perform calculations that are likely to affect health care decisions. Includes for example apps for use by health professionals or users to calculate parameters pertaining to care, such as early warning system software [45].
Diagnose	Health apps that use data to diagnose a health issue in a person, or to guide a diagnostic decision made by a health professional. Includes for example apps that diagnose specified health issues using clinical data [45].

Medical society guidance

[Link to (for this type of health app) relevant European or alternatively European national medical society guidelines (section), recommendations or other type of guidance.]

This link provides guidelines from [Medical society name] that health professionals can use in their decision-making on recommending this type of app to an individual patient.

Health and societal benefit

(Health) interventions to achieve the health benefit

For further development, e.g.:

a. Health intervention(s) (for consideration using ICHI: https://mitel.dimi.uniud.it/ichi/)
b. Behavior change interventions (e.g. education, training, enablement): https://implementationscience.biomedcentral.com/articles/10.1186/1748-5908-6-42/tables/1)
c. Technology-specific interventions (e.g. monitoring symptoms, reminders to promote treatment adherence)
d. Algorithms that can [not] change through learning during use with [automated / human] interpretation
e. Generative AI (e.g. chatbot)
f. Other

- **Not** communicated which health interventions are used to achieve the health benefit
- No health interventions used in the app

- Financial costs to achieve the health benefit communicated
  List financial costs, e.g. data usage, in-app purchases, services, other products needed, in case of optional costs the added benefit, if the financial costs are in part otherwise covered the revenue model
- **Not** communicated if financial costs to achieve the health benefit
- No financial costs to achieve the health benefit

- Health professional support needed to achieve the health benefit communicated
  [Type health professional] [Type support] [Realistic workload] [If need be further details such as use context]
- **Not** communicated if health professional support is needed to achieve the health benefit
- No need for health professional support to achieve the health benefit

- Health benefit evidence of the app
  Detailed PICO analysis for the 5 most relevant papers (clinical studies/real world evidence reports) for the app or related pathway: [Title article/report] [Link to the article/report (not behind a paywall, not just the abstract)] [Study design (e.g. Randomized Controlled Trial, Pre-Post study)] [Peer review (yes/no)] [Population/population (ICD-10 description (3- or 4-digit ICD-10 code) /problem] [Intervention (this health app or demonstrably equivalent)] [Comparator (e.g. standard of care, non-treatment, treatment with a relevant and acknowledged other health app or digital health technology)] [Outcome measures (number, validated yes/no)] [Patient reported outcomes (number, validated yes/no)] [Country/Countries of study (with in footnote rationale of applicability to other countries or regions)] [Number of participants] [Follow-up (time indicator)] [Number of dropouts (with in footnote main reason)] – [Number of clinics / centers involved] and for consideration: [Blinding (yes/no, likely most often not feasible)] [Involvement manufacturer in study (yes/no, could under the right conditions be an advantage, although perhaps not (yet) seen as such)]
- No health benefit evidence needed for this type of app

if health benefit evidence:

- Health benefit evidence includes peer-reviewed research with this app
- **Not** enough evidence that health benefit evidence includes peer-reviewed research with this app

if health benefit evidence:

- Appropriate evidence level for the health benefit
  The level of evidence for the health benefit is appropriate. Based on the NICE Evidence Standards Framework appropriate level of evidence depends on the intended use of an app[1]:

[1] We recommend to further explore the differences between apps and “pills“ as to e.g. appropriate evidence level, evidence details to display (for which we used now PICO as a basis) and appropriate

Apps with an intended use of	Appropriate evidence
Treat, Active monitoring, Calculate and/or Diagnose	Randomized Controlled Trial (RCT) or systematic review or meta-analysis of RCT's
Only Research	An ethics review and approval or an official exemption or waiver. Publishing the research protocol is good research practice.
Preventative behaviour change and/or Self-manage, that do not have an intended use of Treat, Active monitoring, Calculate and/or Diagnose	An observational study. A quasi-experimental study, experimental study, RCT or Systematic review or meta-analysis of RCT's improves the level of evidence.

- **Not** enough evidence of appropriate evidence level for the health benefit

- Societal benefit evidence of the app
  Links to the at most 5 most relevant papers for the app or related pathway
- **Not** enough evidence for societal benefit evidence of the app

- Societal benefit evidence includes peer-reviewed research with this app
- **Not** enough evidence that the societal benefit evidence includes peer-reviewed research with this app

Safety

- Restrictions intended users communicated (see intended users)
- **Not** communicated what restrictions to intended users are
- No restrictions

- Medical device status of the app has been assessed and addressed
  The EU Medical Device Regulation (MDR) is not applicable for this type of app / Self-certified by the manufacturer as [MDR/MDD] class I medical device software / Certified as [MDR/MDD] class [IIa/IIb/III] medical device software by notified body [CE 1234] / if applicable Accessory/ies used with this app is/are self-certified (CE-marked) by the manufacturer as an [MDR/MDD] class I medical device / Accessory/ies used with this app is/are certified (CE-marked) as an [MDR/MDD] class [IIa/IIb/III] medical device by notified body [CE 1234] / Accessory/ies used with this app should be certified (CE marked) as a medical device / registration(s) in EUDAMED

- Health professionals were involved in the development of the app
  If consent: [Names health professionals] [Qualifications] [Professional roles] [Name institution(s)] [Country / Countries (with in footnote rationale as to applicability to more countries)] [Extent of involvement beyond tokenism based on the participation ladder]
- **Not** enough evidence that health professionals were involved in the development of the app

- Peer-reviewed scientific literature was used in the development of the app
  A link to the at most 5 most relevant papers / guidelines
- **Not** enough evidence that peer-reviewed literature was used in the development of the app
- No peer-reviewed scientific literature needed for this type of app

- Health risks of using the app have been analyzed

taxonomies in health interventions. E.g. for the intended use diagnosis, instead of an RCT appropriate evidence could be precision and accuracy validation and comparative studies to prove clinical utility.

- Control measures for identified health risks are in place

- Residual health risks of using the app are found acceptable
  Overview of: [Health risks] [Control measures] [Residual health risks] [Statement how / why the benefit outweighs the residual risks, which includes a comparison with existing / competitive products]

- Potential customers and users are made aware of the health risks, contra-indications and limitations of use of the app

- Option to report safety issue of the app to enable the manufacturer to resolve it
  Link to report a safety issue. Set up may be inspired by the EPREL reporting mechanism for the EU Energy label.
- **Not** enough evidence that safety issues of the app are collected and resolved
- No need to collect safety issues for this type of app

Certifications (for consideration)
[Number e.g. ISO 13485 and full name ISO or other recognized standards]

- Ethical challenges of the app are assessed with users and professionals
- **Not** enough evidence that ethical challenges of the app are assessed with users and professionals

- Ethical approval of the app by an ethics board or advisor
  Copy of approval or exemption documentation
- **Not** enough evidence that the app is approved by an ethics board or advisor

- Health information in the app is maintained
- **Not** enough evidence that the health information in the app is maintained

- Health information sources are all disclosed
  List of health information sources
- **Not** enough evidence that all health information sources are disclosed in the app

- Funding sources are all disclosed
  [List of funding sources and possible conflicts of interest of the app] [Revenue model of the health app if no financial costs to the user]
- **Not** enough evidence that all funding sources of the app are disclosed

- Use of advertising in the app is disclosed and can be declined
- **Not** enough evidence that the use of advertising in the app is disclosed and can be declined
- No use of advertising in the app

Easy to use

Easy to use				
E	D	C	B	A

Accessibility

Accessibility statement of the app
Link to the accessibility statement (https://www.w3.org/WAI/planning/statements/generator/#create)
No accessibility statement available for the app

- Accessibility standard is implemented (e.g. WCAG)
- **Not** enough evidence that accessibility standard is implemented (e.g. WCAG)

if not implemented accessibility standard:
- Measures for all intended users to perceive the app content
- **Not** enough evidence of measures for all intended users to perceive the app content

if not implemented accessibility standard:
- Measures for all intended users to operate the app
- **Not** enough evidence of measures for all intended users to operate the app

if not implemented accessibility standard:
- Measures for all intended users to understand the app content
- **Not** enough evidence of measures for all intended users to understand the app content

Support to install and learn how to use apps is recommended for persons with limited digital experience and/or health literacy. Also in case an app has measures for all intended users to understand the app.

- Age-appropriate app
- **Not** enough evidence that the app is age-appropriate

Usability

- App design is based on research to understand user needs
- **Not** enough evidence that app design is based on research to understand user needs

- Intended users were involved in development of the app
- **Not** enough evidence that intended users were involved in development of the app

- App design refined with usability testing with intended users
- **Not** enough evidence that app design is refined with usability testing with intended users

- Measures in the app to avoid misuse
- **Not** enough evidence of measures in the app to avoid misuse

- Product information for the app
  Link to the product information
- **Not** enough evidence of product information for the app

- Instructions for use for the app
  Link to the instructions for use
- **Not** enough evidence of instructions for use for the app

- User support for the app
  Link to the user support
- **Not** enough evidence of user support for the app

- Option to give usability feedback to improve the app
  Link to the usability feedback
- **Not** enough evidence that usability feedback is collected to improve the app

Secure data

Privacy

Health data processed by the app
List of health data processed
No health data processed by the app

Other personal data processed by the app
List of other personal data (non-health related personal data under GDPR) processed
No other personal data processed by the app

if personal data processed:
- Data minimization is applied

if personal data processed:
- Policy to review and erase data

if personal data processed:
- Privacy statement is available to customers and users
  Link to the privacy statement

if personal data processed:
- Privacy statement summary is available
- **Not** enough evidence that a privacy statement summary is available

if personal data processed:
- Contracts with all data processors

if personal data processed:

Country where data is stored
Country / list of countries where data is stored

if personal data processed:
- Consent is asked for sharing personal data with third parties
- **Not** enough evidence that consent is asked for sharing personal data with third parties

- No personal data is shared with third parties[2]

if personal data processed:

Cookie policy
Link to the cookie policy

if personal data processed:

- Data protection officer for the app
  Role-based e-mail address
- **Not** enough evidence of a data protection officer

if personal data processed:

- Security incident response procedure
- **Not** enough evidence of a security incident response procedure

Security

if personal data processed:

- Security standard is implemented (e.g. ISO 27001)
- **Not** enough evidence that a security standard is implemented (e.g. ISO 27001)

- Security risk impact is assessed
- **Not** enough evidence that security risk impact is assessed

- Secure by design process
- **Not** enough evidence of a secure by design process

- Reliability of third-party software is ensured
- **Not** enough evidence that reliability of third-party software is ensured

- Access to the source code is protected
- **Not** enough evidence that access to the source code is protected

if personal data processed:

- Organizational security measures
- **Not** enough evidence of organizational security measures

if personal data processed:

- Access to the app is secured
- **Not** enough evidence that access to the app is secured

if personal data processed:

- Data is encrypted
- **Not** enough evidence that data is adequately encrypted

- Option to report security vulnerability for the manufacturer to adequately address

[2] Preferably established by test

Link to report a security vulnerability

- **Not** enough evidence that the manufacturer adequately addresses security vulnerabilities

- Security is tested regularly and at major changes
- **Not** enough evidence that security is tested regularly and at major changes

- Data security policy is available to customers and users
  Link to the data security policy
- **Not** enough evidence that the data security policy is available to customers and users

Robust build

Robust build
A

Software robustness

- All product requirements are documented
- **Not** enough evidence that all product requirements are documented

- Developed with software standards, methods and tools (e.g. IEC 62304)
- **Not** enough evidence of development app with software standards, methods and tools (e.g. IEC 62304)

- Secure coding standard is followed
- **Not** enough evidence that a secure coding standard is followed

- Configuration management plan is established
- **Not** enough evidence that a configuration management plan is established

- Process to deal with an increase in demand
- **Not** enough evidence of a process to deal with an increase in demand

- Validation and verification plan is used
- **Not** enough evidence that a validation and verification plan is used

- Release and deployment process is established
- **Not** enough evidence that a release and deployment process is established

- Maintenance process is established
- **Not** enough evidence that a maintenance process is established

Interoperability

- Interoperability standards used
  List of interoperability standards used with for consideration link to specification / implementation guidelines
- **Not** enough evidence that the specifications for APIs can be accessed

- No connection with an electronic health record (EHR) or other devices

if APIs:

European EHR exchange format is supported
Not enough evidence that the European EHR exchange format is supported

if APIs:

- Terminologies used
  List of terminologies used with for consideration link to specification / implementation guidelines
- **Not** enough evidence that the specifications for terminologies can be accessed

if APIs:

- All data from other sources is validated before use
- **Not** enough evidence that **all** data from other sources is validated before use
- No data from other sources imported

if personal data processed:

- Personal data can be exported to another platform
- **Not** enough evidence that personal data can be exported to another platform
- No new data to export to another platform
