# Supplementary material for: Developing a Health App Quality Report for CEN-ISO/TS 82304-2 to Satisfy Health Care Professionals’ Information Needs on the Quality of Health Apps: Participatory Design Study
Source: J Particip Med. 2026 Oct 23;18:e86473. doi: 10.2196/86473 (PMC13693862; doi:10.2196/86473)
Supplement: Supplementary file 8 [file jopm-v18-e86473-s008.pdf]

Multimedia Appendix 8. Comparative Content Analysis

Legend: (white) = not provided; (lightest blue) = 0 clicks; (light blue) = 1 click; (medium blue) = 2 clicks; (dark navy) = >2 clicks

Quality report<br># of digital health products displayed<br><br>Type detail information	82304-2<br>test phase<br>(August 2024)	DiGA[ab]<br>64 apps<br>(August 2024)	Digi-HTA[ab]<br>15 products<br>(August 2024)	AppGuide[a]<br>322.020 apps<br>(May 2024)
App name	0 clicks	0 clicks	0 clicks	0 clicks
App icon	0 clicks	0 clicks	not provided	0 clicks
Platforms	0 clicks	0 clicks	1 click	[c] 1 click
Manufacturer	0 clicks	0 clicks	1 click	0 clicks
Intended users	0 clicks	0 clicks	2 clicks	2 clicks
Intended use	0 clicks	1 click	0 clicks	1 click
Offline use supported	0 clicks	2 clicks	2 clicks	not provided
Overall health app quality score	0 clicks	not provided	0 clicks	not provided
Date app (last) checked	0 clicks	1 click	0 clicks	1 click
Score Healthy and safe	0 clicks	not provided	1 click	1 click
Score Easy to use	0 clicks	not provided	1 click	1 click
Score Secure data	0 clicks	not provided	1 click	1 click
Score Robust build	0 clicks	not provided	not provided	not provided
Pass / fail individual requirements	1 click	2 clicks	not provided	2 clicks
Manufacturer contact details	1 click	[d] 2 clicks	[d] 2 clicks	2 clicks
Devices supported (computer, ..)	1 click	2 clicks	1 click	not provided
Languages	1 click	0 clicks	1 click	1 click
Details app version (checked)	1 click	[e] 2 clicks	not provided	1 click
Assessment validity period[f]	1 click	1 click	0 clicks	not provided
App assessment methods	1 click	2 clicks	2 clicks	>2 clicks
European EHRxF[g] supported	1 click	not provided	not provided	not provided
Details intended users	2 clicks	2 clicks	2 clicks	not provided
Contra-indications	2 clicks	2 clicks	2 clicks	not provided
Details intended use	2 clicks	2 clicks	1 click	not provided
Medical society guidance	2 clicks	2 clicks	[h] 2 clicks	not provided
Health interventions	2 clicks	2 clicks	2 clicks	[i] 1 click
Additional cost / accessories	2 clicks	2 clicks	2 clicks	1 click
HCP support needed	2 clicks	2 clicks	2 clicks	not provided
Health benefit evidence	2 clicks	2 clicks	2 clicks	2 clicks
Evidence level information	2 clicks	2 clicks	[j] not provided	not provided
Societal benefit evidence	2 clicks	2 clicks	2 clicks	not provided
Medical device status	2 clicks	2 clicks	1 click	not provided
Developed with HCPs	2 clicks	2 clicks	not provided	not provided
Developed with literature	2 clicks	2 clicks	2 clicks	not provided
(Residual) health risks, controls	2 clicks	not provided	2 clicks	not provided
Option to report safety issue	2 clicks	not provided	not provided	not provided
Certifications (for consideration)	2 clicks	not provided	not provided	[k] >2 clicks

Ethical approval				
Health information sources				
Funding sources				
Accessibility statement				
Product information (app website)				
Instructions for use (details on)				
User support				
Option to give usability feedback			l	
Health data processed				
Other personal data processed				
Privacy statement				
Country where data is stored				
Cookie policy				
DPO contact details				
Option to report sec. vulnerability				
Data security policy available			m	
Interoperability standards			n	
Terminologies used				
Separate information for HCPs				
Adherence / sustained use details				
Suppliers (eg cloud service)				
Data retention details				
Data portability details				
Downtime details				
Assessment team				
Screenshots				
Verify language/country (search)				
Last update				
Countries app available				
Recommendation organization				
Star ratings and # downloads				
Which privacy law compliant				

DPO = Data Protection Officer, EEHRxF = European electronic health record exchange format, HCP = health care professional

[a] Based on the first 2 apps published in the registry, DiGA: Actensio, Cara Care für Reizdarm, Digi-HTA: Terve Päivä, Tunstall TSP, AppGuide: Abs workout A6W - flat belly, AbleTo

[b] Includes context specific details such as DiGA: status (provisionally included, permanently included, cancelled), costs, classification in the care pathway, prescription procedures, Digi-HTA: costs, guidance on procurement, current customers (health providers, health authorities)

[c] Link to download

[d] Link to “about page” on the company website

[e] More detailed, includes compatibility details

[f] CEN-ISO/TS 82304-2 publishes according to its certification scheme having used the assessment guidelines of the calendar year. As (regulatory) context, technology, notions on what is proportional assessment, assessment methods, etc. change, intent is to update these guidelines on an annual basis and more frequent in case of urgent matters. Also, applicable evidence has to be reassessed whenever evidence for a requirement expires, and when the app has a new release with changes that

affect requirement, aspect and overall scores or content of the report. DiGA makes a distinction in provisionally included, permanently included, and cancelled with related dates. Digi-HTA publishes a validity of 3 years.

[g] For more information on the European electronic health record exchange format see https://digital-strategy.ec.europa.eu/en/policies/electronic-health-records

[h] Literature reviews, recommendations and previous (HTA-)assessments for this type of app

[i] Distinction in data oriented and care oriented features

[j] Can be established via link to article

[k] Declared compliances to security certification and design standard

[l] Manufacturer usability review (app 1)

[m] Risk management and data security testing details, log management details, user management (authentication) details, hardware, data protection, other things to note (app 2 only): ISO/IEC 27001:2013 certification

[n] Instead of details on interoperability standards, details on interoperability with Electronic Health Record system / other health and wellbeing devices
