# Supplementary material for: Developing a Health App Quality Report for CEN-ISO/TS 82304-2 to Satisfy Health Care Professionals’ Information Needs on the Quality of Health Apps: Participatory Design Study
Source: J Particip Med. 2026 Oct 23;18:e86473. doi: 10.2196/86473 (PMC13693862; doi:10.2196/86473)
Supplement: Supplementary file 9 [file jopm-v18-e86473-s009.pdf]

Multimedia Appendix 9. Health Education Materials Assessment Tool (HEMAT) Results

= no = in part = yes

	82304-2	DiGA[a]	Digi-HTA[a]	AppGuide[a]
Obvious main message	yes	yes	yes	yes
All information needed, not overwhelming	yes	in part	no	no
Common everyday language	in part	no	no	yes
Mostly active voice	no	no	no	no
Information needed easily found	yes	no	in part	yes
Visual cues make takeaways clear	yes	no	in part	yes
Visual aids support main message	in part	no	no	in part
Numbers are easy to understand	yes	no [b]	no [b]	N.A.

N.A. = not applicable

[a] Based on the first 2 apps published in the registry, DiGA: Actensio, Cara Care für Reizdarm, Digi-HTA: Terve Päivä, Tunstall TSP, AppGuide: Abs workout A6W - flat belly, AbleTo

[b] Statistical analysis
